# Supplementary material for: Bidirectional relationship between olfaction and Parkinson’s disease
Source: NPJ Parkinsons Dis. 2024 Dec 5;10:232. doi: 10.1038/s41531-024-00838-4 (PMC11621548; doi:10.1038/s41531-024-00838-4)
Supplement: Supplementary file 1 — Supplementary Information [file 41531_2024_838_MOESM1_ESM.pdf]

a.

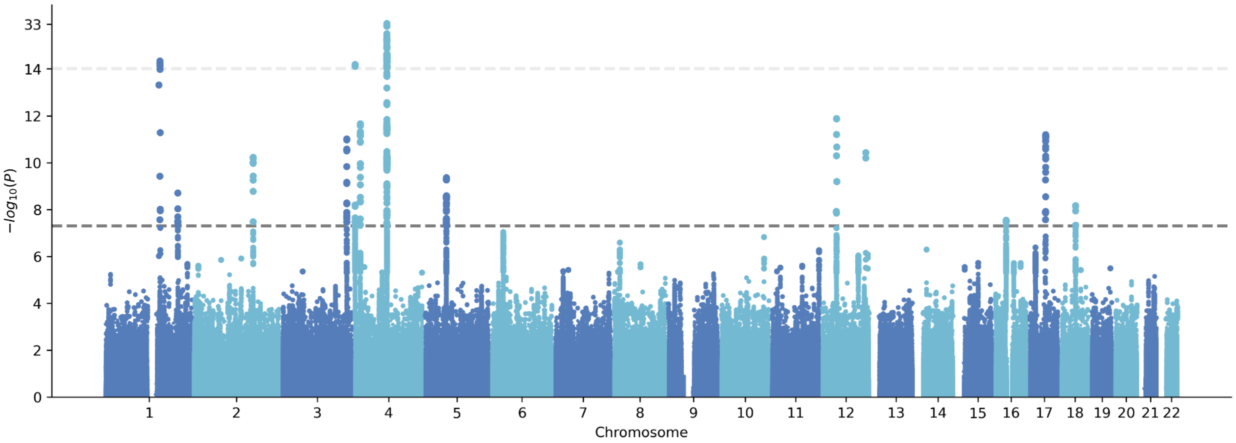

b.

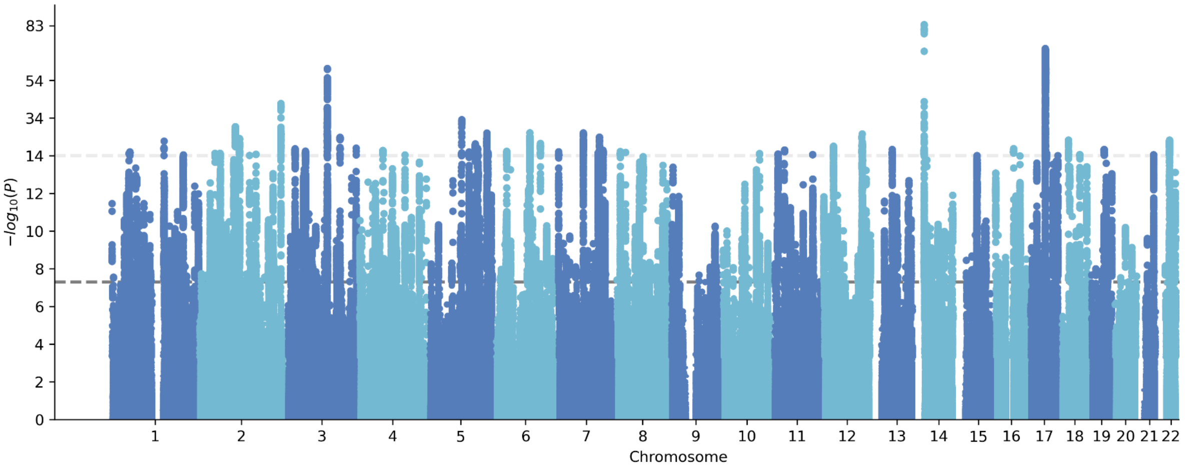

Supplementary Figure 1 - Manhattan Plots of a) PD and b) ability to smell GWAS. Y-axis represents the negative log two-tailed *p*-value of association for the individual trait, while the x-axis represents the chromosome-base pair positions of the SNPs.

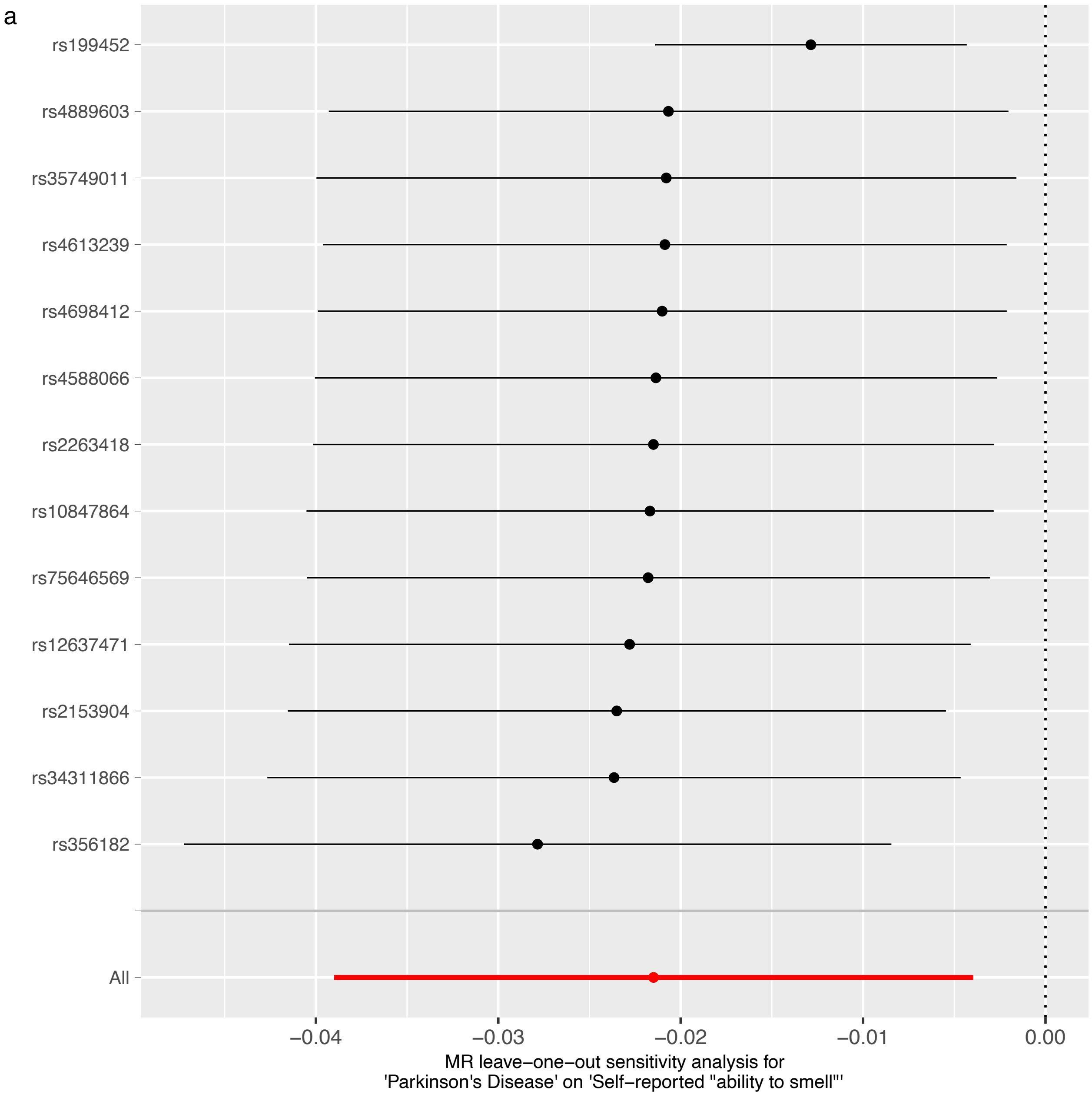
a
rs199452
rs4889603
rs35749011
rs4613239
rs4698412
rs4588066
rs2263418
rs10847864
rs75646569
rs12637471
rs2153904
rs34311866
rs356182
All
−0.04
−0.03
−0.02
−0.01
0.00
MR leave−one−out sensitivity analysis for
'Parkinson's Disease' on 'Self−reported "ability to smell"'

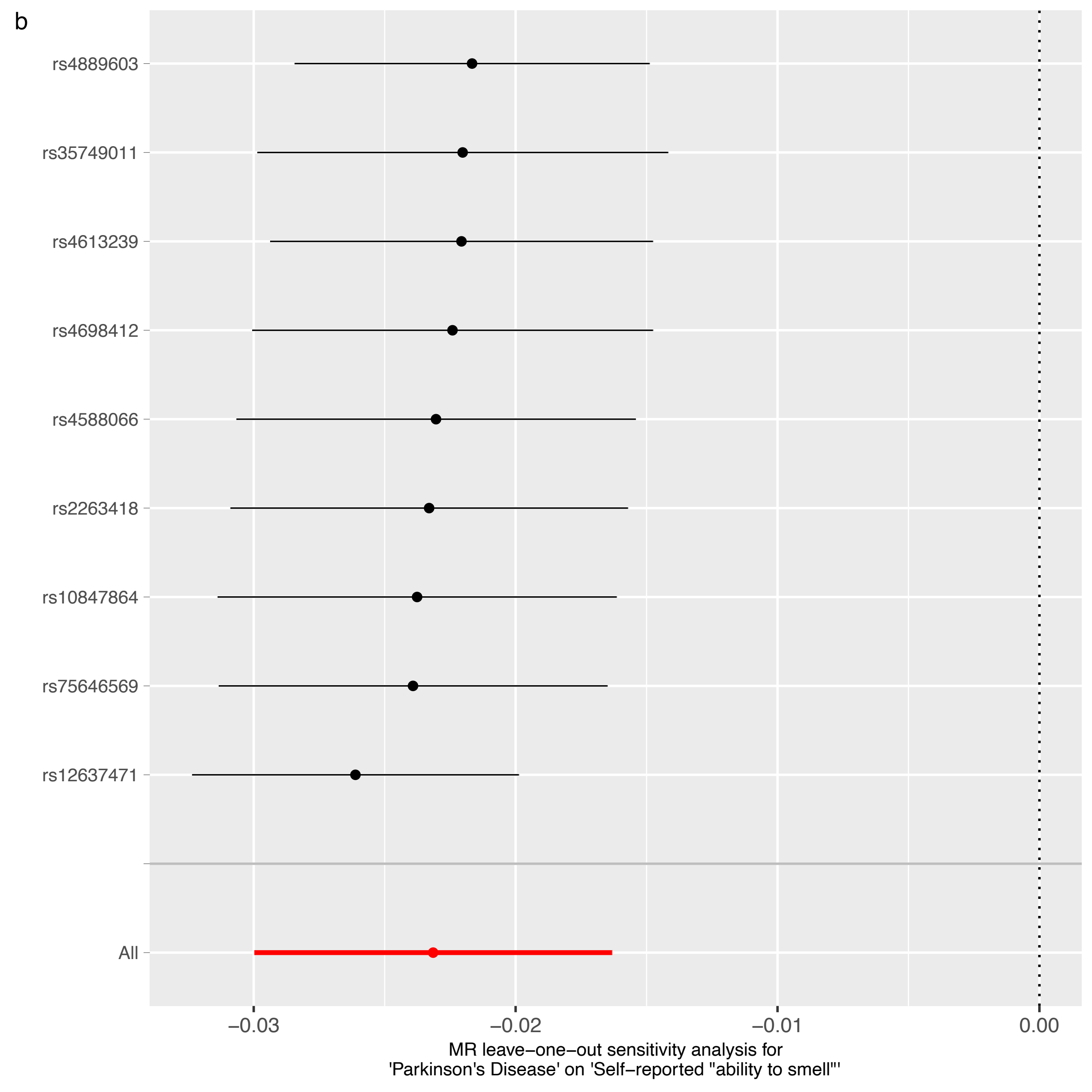
b
rs4889603
rs35749011
rs4613239
rs4698412
rs4588066
rs2263418
rs10847864
rs75646569
rs12637471
All
−0.03
−0.02
−0.01
0.00
MR leave−one−out sensitivity analysis for
'Parkinson's Disease' on 'Self−reported "ability to smell"'

c

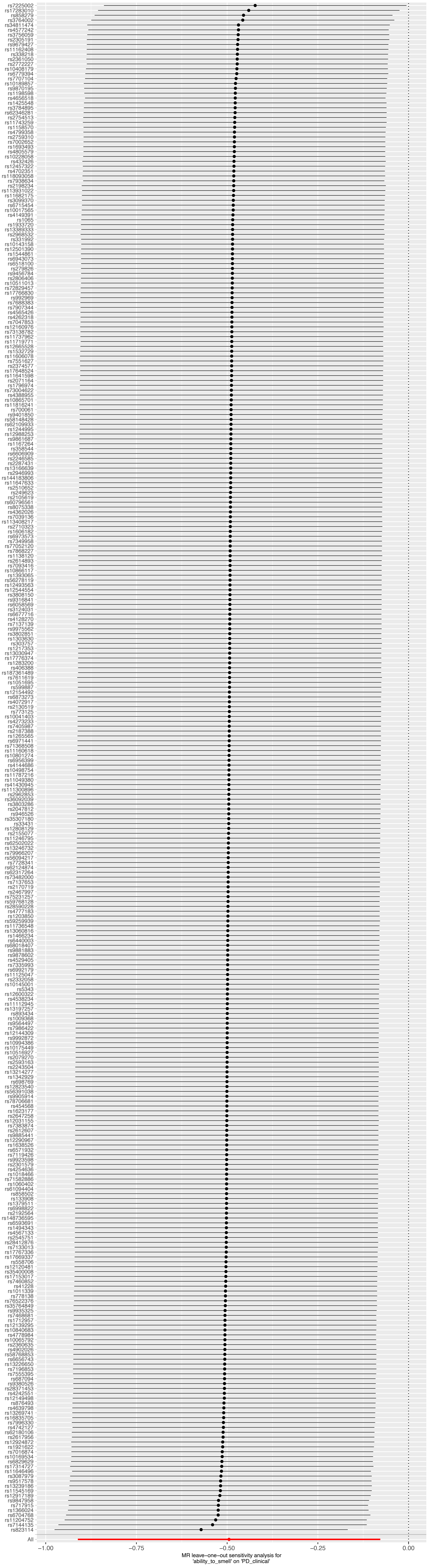
All
−1.00
−0.75
−0.50
−0.25
0.00
MR leave−one−out sensitivity analysis for
'ability_to_smell' on 'PD_clinical'

d

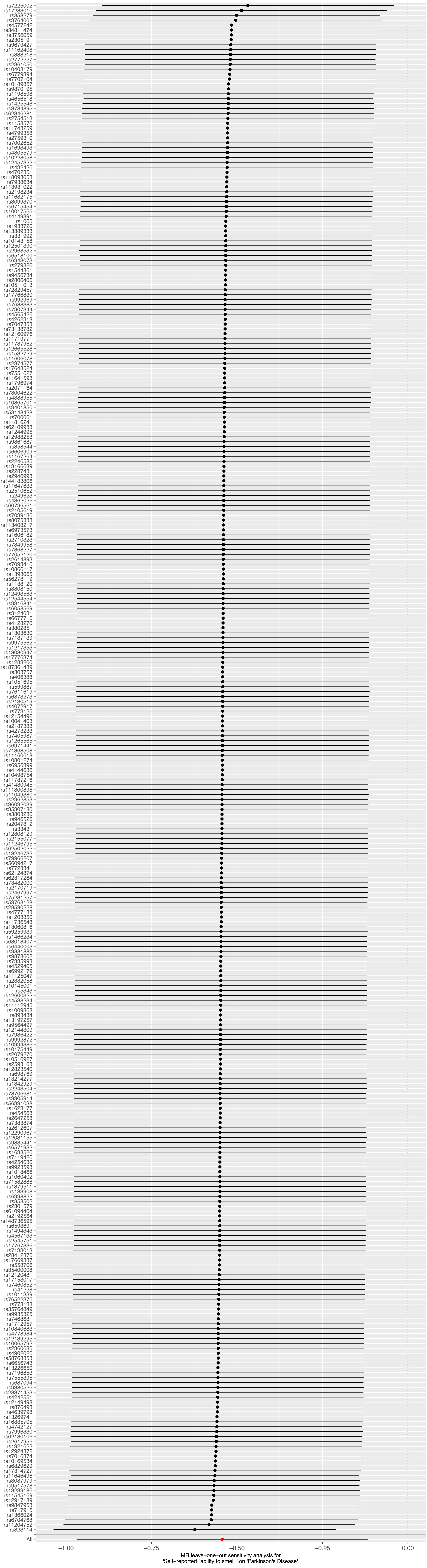
All
−1.00
−0.75
−0.50
−0.25
0.00
MR leave−one−out sensitivity analysis for
'Self−reported "ability to smell"' on 'Parkinson's Disease'

Supplementary Figure 2 - Forest plot of leave-one-out sensitivity results for a) PD on ability to smell b) PD on ability to smell with MR-PRESSO outliers removed c) ability to smell on PD d) ability to smell on PD with MR-PRESSO outliers removed. The SNP removed for the leave-one-out result is indicated on the y-axis, while the result at the bottom indicates the IVW results without any SNPs removed. Horizontal bar indicates the 95% confidence interval for the effect size in log odds ratio.

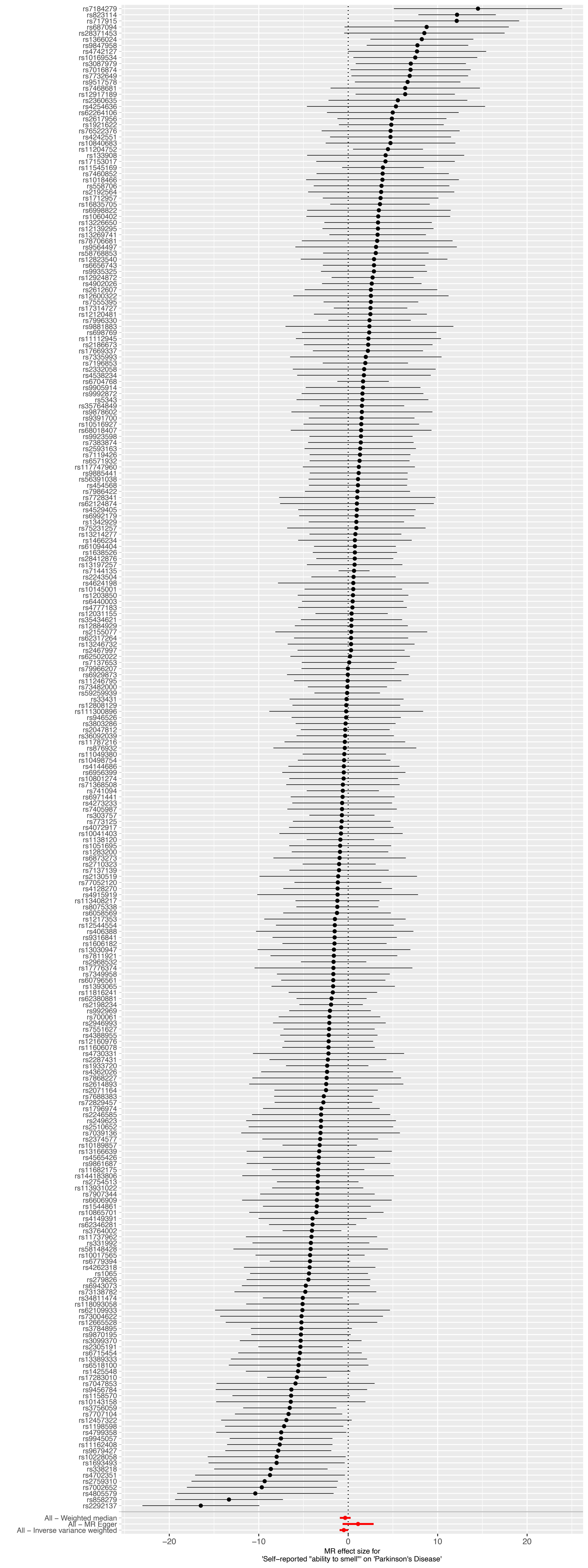
All – Weighted median
All – MR Egger
All – Inverse variance weighted
−20
−10
0
10
20
MR effect size for
'Self-reported "ability to smell"' on 'Parkinson's Disease'

Supplementary Figure 3 - Forest plot of ability to smell on PD MR results. The individual SNP MR results are colored in black while the meta-analysis results are in red. The y-axis label indicates the individual SNPs for the single SNP MR or the meta-analysis method for the multi-SNP MR. The x-axis represents the MR effect size in log odds ratio. The horizontal bars indicate the 95% confidence interval for the MR effect size.

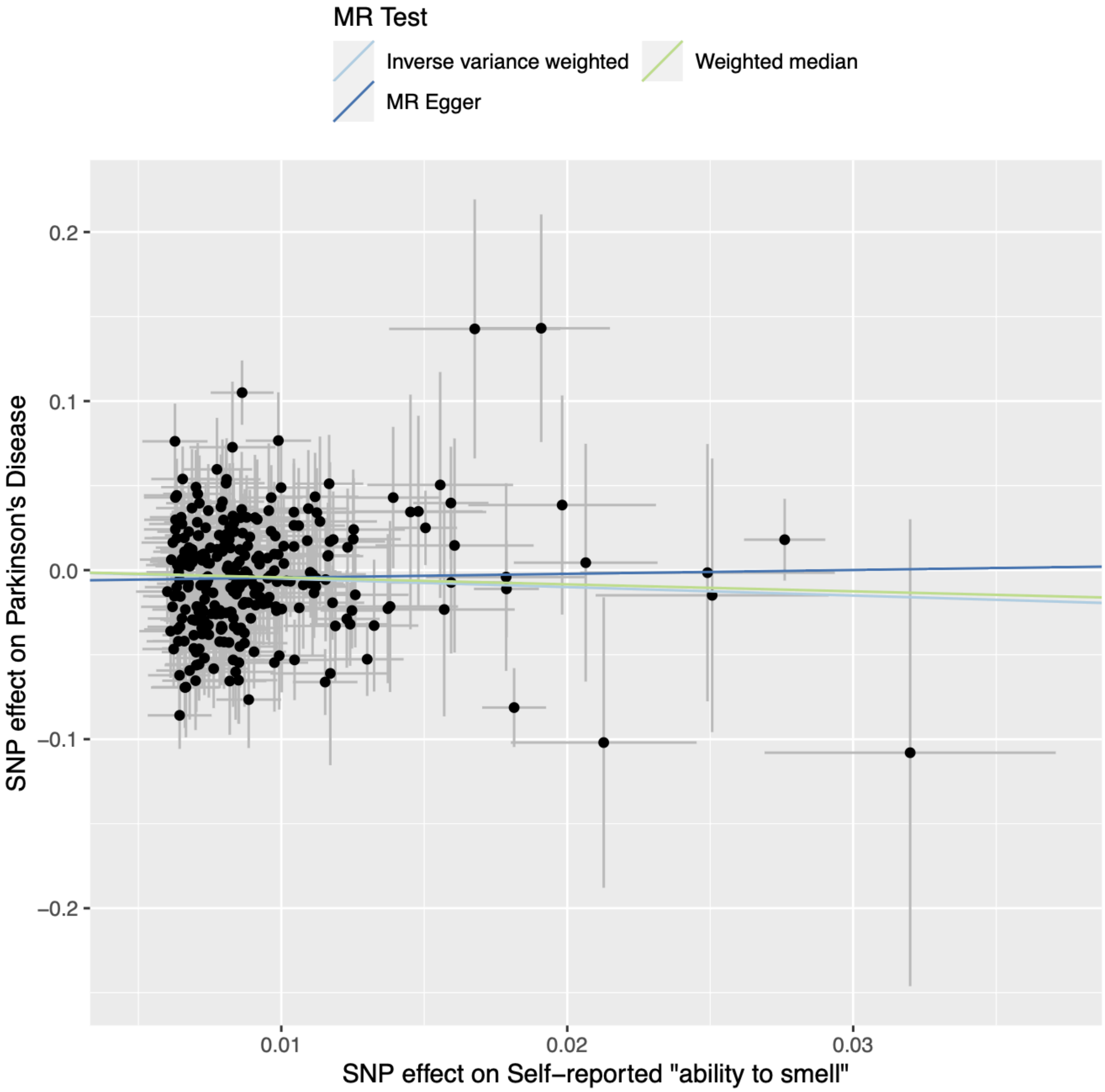

Supplementary Figure 4 - Scatter plot of ability to smell on PD MR results. The y and x-axes indicate the effect size of SNPs on PD and the ability to smell respectively in log odds ratio. The vertical and horizontal bars indicate the 95% confidence interval for the effect size on the ability to smell and PD risk respectively. Colored lines represent the different MR results.

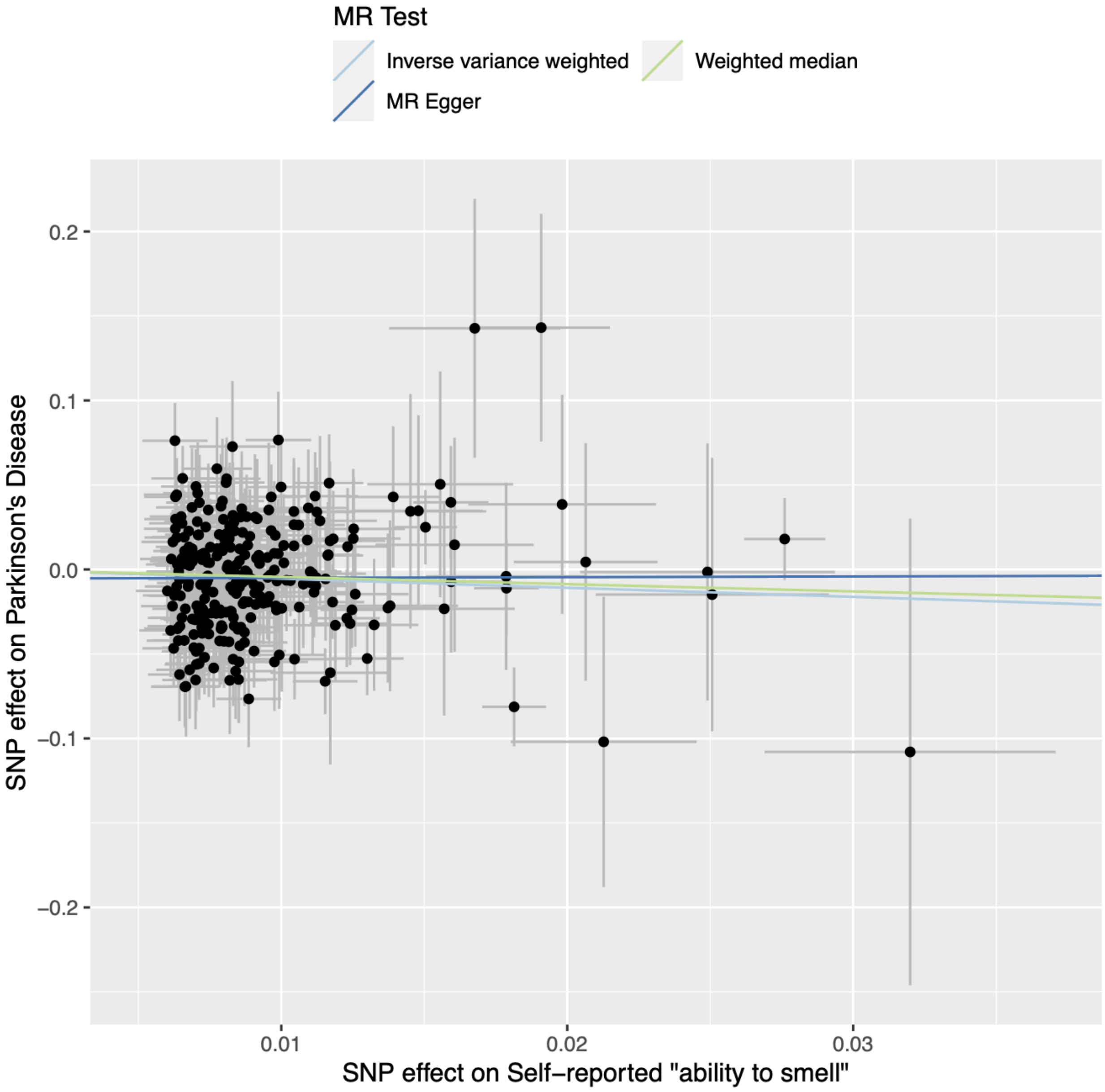

Supplementary Figure 5 - Scatter plot of ability to smell on PD MR results with MR-PRESSO outliers removed. The y and x-axes indicate the effect size of SNPs on PD risk and the ability to smell respectively in log odds ratio. The vertical and horizontal bars indicate the 95% confidence interval for the effect size on the ability to smell and PD risk respectively. Colored lines represent the different MR results.

Supplementary Table 1 – GWAS Descriptions. heritability (h2) is in liability scale for PD and observed scale for ability to smell.

Trait	Number of loci	Genomic Lambda (GC)	h2 (se)	LD score intercept
PD	13	1.0625	0.1035 (0.0111)	0.9737
Ability to smell	394	2.0715	0.0533 (0.0016)	1.0637

Supplementary Table 2 – MR Heterogeneity test. *Q* – Cochran's Q; df – degrees of freedom; P_Q – *p*-value of heterogeneity test; I^2 – I-squared statistic.

Exposure	Outcome	Method	*Q*	df	P_Q	I^2
Parkinson's Disease	Ability to smell	Inverse variance weighted	210.2	12	2.52E-38	94.3
Parkinson's Disease	Ability to smell	MR Egger	205.4	11	5.63E-38	94.6
Parkinson's Disease - outliers removed	Ability to smell	Inverse variance weighted	11.0	8	0.202	27.2
Parkinson's Disease - outliers removed	Ability to smell	MR Egger	11.0	7	0.139	36.3
Ability to smell	Parkinson's Disease	Inverse variance weighted	417.0	275	6.68E-08	34.1
Ability to smell	Parkinson's Disease	MR Egger	415.6	274	6.93E-08	34.1
Ability to smell - outliers removed	Parkinson's Disease	Inverse variance weighted	351.6	271	0.000692	22.9
Ability to smell - outliers removed	Parkinson's Disease	MR Egger	351.2	270	0.000632	23.1

Supplementary Table 3 – Egger intercept test for pleiotropy. SE: standard error of the Egger intercept; P: *p*-value

Exposure	Outcome	Egger intercept	SE	P
Parkinson's Disease	Ability to smell	-0.002	0.005	0.621
Parkinson's Disease - LAVA region removed	Ability to smell	-0.006	0.003	0.123
Parkinson's Disease - outliers removed	Ability to smell	0.000	0.002	0.977
Ability to smell	Parkinson's Disease	-0.007	0.007	0.332
Ability to smell - LAVA region removed	Parkinson's Disease	-0.007	0.006	0.236
Ability to smell - outliers removed	Parkinson's Disease	-0.004	0.007	0.570

Supplementary Table 4 – IVW Leave-one-out sensitivity test results. SNP removed: rsID of the SNP removed for the leave-one-out test; BETA: effect size in logOR scale; SE: standard error of effect size; P: *p*-value of the leave-one-out test.

**Exposure**	**Outcome**	**SNP removed**	**BETA**	**SE**	**P**
Parkinson's Disease	Ability to smell	rs10847864	-0.022	0.010	0.024
Parkinson's Disease	Ability to smell	rs12637471	-0.023	0.010	0.017
Parkinson's Disease	Ability to smell	rs199452	-0.013	0.004	0.003
Parkinson's Disease	Ability to smell	rs2153904	-0.024	0.009	0.011
Parkinson's Disease	Ability to smell	rs2263418	-0.021	0.010	0.024
Parkinson's Disease	Ability to smell	rs34311866	-0.024	0.010	0.015
Parkinson's Disease	Ability to smell	rs356182	-0.028	0.010	0.005
Parkinson's Disease	Ability to smell	rs35749011	-0.021	0.010	0.034
Parkinson's Disease	Ability to smell	rs4588066	-0.021	0.010	0.025
Parkinson's Disease	Ability to smell	rs4613239	-0.021	0.010	0.029
Parkinson's Disease	Ability to smell	rs4698412	-0.021	0.010	0.029
Parkinson's Disease	Ability to smell	rs4889603	-0.021	0.009	0.030
Parkinson's Disease	Ability to smell	rs75646569	-0.022	0.010	0.023
Parkinson's Disease - outliers removed	Ability to smell	rs10847864	-0.024	0.004	9.57E-10
Parkinson's Disease - outliers removed	Ability to smell	rs12637471	-0.026	0.003	2.18E-16
Parkinson's Disease - outliers removed	Ability to smell	rs2263418	-0.023	0.004	1.72E-09
Parkinson's Disease - outliers removed	Ability to smell	rs35749011	-0.022	0.004	3.65E-08
Parkinson's Disease - outliers removed	Ability to smell	rs4588066	-0.023	0.004	3.06E-09
Parkinson's Disease - outliers removed	Ability to smell	rs4613239	-0.022	0.004	3.21E-09
Parkinson's Disease - outliers removed	Ability to smell	rs4698412	-0.022	0.004	9.35E-09
Parkinson's Disease - outliers removed	Ability to smell	rs4889603	-0.022	0.003	3.63E-10
Parkinson's Disease - outliers removed	Ability to smell	rs75646569	-0.024	0.004	2.60E-10
Ability to smell	Parkinson's Disease	rs10017565	-0.483	0.213	0.023
Ability to smell	Parkinson's Disease	rs10041403	-0.494	0.213	0.020
Ability to smell	Parkinson's Disease	rs10065792	-0.506	0.212	0.017
Ability to smell	Parkinson's Disease	rs1009368	-0.500	0.213	0.019

Ability to smell	Parkinson's Disease	rs1011339	-0.505	0.213	0.018
Ability to smell	Parkinson's Disease	rs10143158	-0.485	0.213	0.023
Ability to smell	Parkinson's Disease	rs10145001	-0.499	0.213	0.019
Ability to smell	Parkinson's Disease	rs10169534	-0.514	0.212	0.015
Ability to smell	Parkinson's Disease	rs10175449	-0.500	0.213	0.019
Ability to smell	Parkinson's Disease	rs1018466	-0.501	0.213	0.018
Ability to smell	Parkinson's Disease	rs10189857	-0.477	0.213	0.025
Ability to smell	Parkinson's Disease	rs10228058	-0.480	0.212	0.024
Ability to smell	Parkinson's Disease	rs10408179	-0.473	0.212	0.026
Ability to smell	Parkinson's Disease	rs10498754	-0.495	0.213	0.020
Ability to smell	Parkinson's Disease	rs10511013	-0.486	0.213	0.022
Ability to smell	Parkinson's Disease	rs10516927	-0.500	0.213	0.019
Ability to smell	Parkinson's Disease	rs1051695	-0.493	0.213	0.021
Ability to smell	Parkinson's Disease	rs1060402	-0.502	0.213	0.018
Ability to smell	Parkinson's Disease	rs1065	-0.485	0.213	0.023
Ability to smell	Parkinson's Disease	rs10801274	-0.495	0.213	0.020
Ability to smell	Parkinson's Disease	rs10840683	-0.506	0.213	0.017
Ability to smell	Parkinson's Disease	rs10865701	-0.489	0.213	0.022
Ability to smell	Parkinson's Disease	rs10866117	-0.492	0.213	0.021
Ability to smell	Parkinson's Disease	rs10994386	-0.500	0.213	0.019
Ability to smell	Parkinson's Disease	rs11049380	-0.495	0.213	0.020
Ability to smell	Parkinson's Disease	rs11112945	-0.500	0.213	0.019
Ability to smell	Parkinson's Disease	rs11125047	-0.499	0.213	0.019
Ability to smell	Parkinson's Disease	rs111300896	-0.495	0.213	0.020
Ability to smell	Parkinson's Disease	rs11160618	-0.495	0.213	0.020
Ability to smell	Parkinson's Disease	rs11162408	-0.471	0.212	0.026
Ability to smell	Parkinson's Disease	rs11204752	-0.532	0.212	0.012
Ability to smell	Parkinson's Disease	rs11246795	-0.496	0.213	0.020
Ability to smell	Parkinson's Disease	rs113408217	-0.491	0.213	0.021
Ability to smell	Parkinson's Disease	rs1138120	-0.492	0.214	0.021
Ability to smell	Parkinson's Disease	rs113931022	-0.482	0.213	0.024
Ability to smell	Parkinson's Disease	rs11545169	-0.519	0.213	0.015
Ability to smell	Parkinson's Disease	rs1158570	-0.479	0.212	0.024
Ability to smell	Parkinson's Disease	rs11606078	-0.488	0.213	0.022
Ability to smell	Parkinson's Disease	rs11641598	-0.488	0.213	0.022
Ability to smell	Parkinson's Disease	rs11646496	-0.516	0.210	0.014
Ability to smell	Parkinson's Disease	rs11647633	-0.490	0.213	0.021
Ability to smell	Parkinson's Disease	rs1167264	-0.490	0.213	0.021
Ability to smell	Parkinson's Disease	rs11682175	-0.483	0.213	0.024
Ability to smell	Parkinson's Disease	rs11719771	-0.487	0.212	0.022

Ability to smell	Parkinson's Disease	rs11736548	-0.498	0.213	0.019
Ability to smell	Parkinson's Disease	rs11737962	-0.487	0.213	0.022
Ability to smell	Parkinson's Disease	rs11743259	-0.479	0.213	0.025
Ability to smell	Parkinson's Disease	rs11787216	-0.495	0.213	0.020
Ability to smell	Parkinson's Disease	rs118093058	-0.482	0.213	0.024
Ability to smell	Parkinson's Disease	rs11816241	-0.489	0.213	0.022
Ability to smell	Parkinson's Disease	rs1198598	-0.478	0.212	0.024
Ability to smell	Parkinson's Disease	rs12031155	-0.501	0.214	0.019
Ability to smell	Parkinson's Disease	rs1203850	-0.498	0.213	0.020
Ability to smell	Parkinson's Disease	rs12120481	-0.503	0.213	0.018
Ability to smell	Parkinson's Disease	rs12139295	-0.506	0.213	0.017
Ability to smell	Parkinson's Disease	rs12144309	-0.500	0.213	0.019
Ability to smell	Parkinson's Disease	rs12149498	-0.508	0.213	0.017
Ability to smell	Parkinson's Disease	rs12154492	-0.494	0.214	0.021
Ability to smell	Parkinson's Disease	rs12160976	-0.487	0.213	0.022
Ability to smell	Parkinson's Disease	rs1217353	-0.493	0.213	0.021
Ability to smell	Parkinson's Disease	rs12290967	-0.501	0.213	0.019
Ability to smell	Parkinson's Disease	rs1244995	-0.490	0.213	0.021
Ability to smell	Parkinson's Disease	rs12457322	-0.481	0.212	0.024
Ability to smell	Parkinson's Disease	rs12493563	-0.492	0.213	0.021
Ability to smell	Parkinson's Disease	rs12501390	-0.485	0.213	0.023
Ability to smell	Parkinson's Disease	rs12544554	-0.492	0.213	0.021
Ability to smell	Parkinson's Disease	rs12600322	-0.499	0.213	0.019
Ability to smell	Parkinson's Disease	rs1265565	-0.494	0.213	0.020
Ability to smell	Parkinson's Disease	rs12665528	-0.487	0.213	0.022
Ability to smell	Parkinson's Disease	rs12808129	-0.496	0.213	0.020
Ability to smell	Parkinson's Disease	rs12823540	-0.500	0.213	0.019
Ability to smell	Parkinson's Disease	rs1283200	-0.493	0.213	0.021
Ability to smell	Parkinson's Disease	rs12917189	-0.520	0.212	0.014
Ability to smell	Parkinson's Disease	rs12924872	-0.512	0.213	0.016
Ability to smell	Parkinson's Disease	rs12988253	-0.490	0.213	0.021
Ability to smell	Parkinson's Disease	rs13030947	-0.493	0.213	0.021
Ability to smell	Parkinson's Disease	rs1303630	-0.493	0.213	0.021
Ability to smell	Parkinson's Disease	rs13060816	-0.498	0.213	0.019
Ability to smell	Parkinson's Disease	rs13166639	-0.490	0.213	0.021
Ability to smell	Parkinson's Disease	rs13197257	-0.500	0.213	0.019
Ability to smell	Parkinson's Disease	rs13214277	-0.500	0.213	0.019
Ability to smell	Parkinson's Disease	rs13226650	-0.507	0.213	0.017
Ability to smell	Parkinson's Disease	rs13239186	-0.518	0.213	0.015
Ability to smell	Parkinson's Disease	rs13246732	-0.497	0.213	0.020

Ability to smell	Parkinson's Disease	rs13269741	-0.509	0.213	0.017
Ability to smell	Parkinson's Disease	rs13389333	-0.485	0.213	0.023
Ability to smell	Parkinson's Disease	rs133908	-0.502	0.213	0.018
Ability to smell	Parkinson's Disease	rs1342929	-0.500	0.213	0.019
Ability to smell	Parkinson's Disease	rs1366024	-0.525	0.211	0.013
Ability to smell	Parkinson's Disease	rs1379511	-0.502	0.213	0.018
Ability to smell	Parkinson's Disease	rs1393065	-0.492	0.213	0.021
Ability to smell	Parkinson's Disease	rs1425548	-0.478	0.213	0.025
Ability to smell	Parkinson's Disease	rs144183806	-0.490	0.213	0.021
Ability to smell	Parkinson's Disease	rs1466234	-0.498	0.213	0.019
Ability to smell	Parkinson's Disease	rs148736595	-0.502	0.213	0.018
Ability to smell	Parkinson's Disease	rs1494343	-0.502	0.213	0.018
Ability to smell	Parkinson's Disease	rs1532729	-0.487	0.213	0.022
Ability to smell	Parkinson's Disease	rs1544861	-0.485	0.213	0.023
Ability to smell	Parkinson's Disease	rs1606182	-0.491	0.213	0.021
Ability to smell	Parkinson's Disease	rs1623177	-0.501	0.213	0.019
Ability to smell	Parkinson's Disease	rs1638526	-0.501	0.213	0.019
Ability to smell	Parkinson's Disease	rs16835705	-0.510	0.213	0.017
Ability to smell	Parkinson's Disease	rs1693493	-0.480	0.212	0.024
Ability to smell	Parkinson's Disease	rs1712957	-0.506	0.213	0.017
Ability to smell	Parkinson's Disease	rs17153017	-0.504	0.213	0.018
Ability to smell	Parkinson's Disease	rs17283010	-0.440	0.212	0.038
Ability to smell	Parkinson's Disease	rs17314727	-0.515	0.213	0.016
Ability to smell	Parkinson's Disease	rs17648524	-0.488	0.213	0.022
Ability to smell	Parkinson's Disease	rs17669337	-0.503	0.213	0.018
Ability to smell	Parkinson's Disease	rs17766830	-0.486	0.213	0.022
Ability to smell	Parkinson's Disease	rs17767336	-0.502	0.213	0.018
Ability to smell	Parkinson's Disease	rs17776374	-0.493	0.213	0.021
Ability to smell	Parkinson's Disease	rs1796974	-0.488	0.213	0.022
Ability to smell	Parkinson's Disease	rs187361489	-0.493	0.213	0.021
Ability to smell	Parkinson's Disease	rs1921622	-0.512	0.213	0.016
Ability to smell	Parkinson's Disease	rs1933720	-0.485	0.213	0.023
Ability to smell	Parkinson's Disease	rs2047812	-0.496	0.213	0.020
Ability to smell	Parkinson's Disease	rs2071164	-0.488	0.213	0.022
Ability to smell	Parkinson's Disease	rs2079270	-0.500	0.213	0.019
Ability to smell	Parkinson's Disease	rs2105619	-0.491	0.213	0.021
Ability to smell	Parkinson's Disease	rs2130519	-0.494	0.213	0.020
Ability to smell	Parkinson's Disease	rs2155077	-0.496	0.213	0.020
Ability to smell	Parkinson's Disease	rs2170719	-0.497	0.213	0.020
Ability to smell	Parkinson's Disease	rs2187388	-0.494	0.213	0.020

Ability to smell	Parkinson's Disease	rs2192564	-0.502	0.213	0.018
Ability to smell	Parkinson's Disease	rs2198234	-0.482	0.214	0.024
Ability to smell	Parkinson's Disease	rs2243504	-0.500	0.213	0.019
Ability to smell	Parkinson's Disease	rs2246585	-0.490	0.213	0.021
Ability to smell	Parkinson's Disease	rs2287431	-0.490	0.213	0.021
Ability to smell	Parkinson's Disease	rs2301579	-0.501	0.214	0.019
Ability to smell	Parkinson's Disease	rs2305191	-0.470	0.213	0.027
Ability to smell	Parkinson's Disease	rs2332058	-0.499	0.213	0.019
Ability to smell	Parkinson's Disease	rs2360635	-0.506	0.213	0.017
Ability to smell	Parkinson's Disease	rs2361050	-0.472	0.212	0.026
Ability to smell	Parkinson's Disease	rs2374577	-0.488	0.213	0.022
Ability to smell	Parkinson's Disease	rs2467997	-0.497	0.213	0.020
Ability to smell	Parkinson's Disease	rs249623	-0.491	0.213	0.021
Ability to smell	Parkinson's Disease	rs2510652	-0.490	0.213	0.021
Ability to smell	Parkinson's Disease	rs2545751	-0.502	0.213	0.018
Ability to smell	Parkinson's Disease	rs2593163	-0.500	0.213	0.019
Ability to smell	Parkinson's Disease	rs2612607	-0.501	0.213	0.019
Ability to smell	Parkinson's Disease	rs2614893	-0.492	0.213	0.021
Ability to smell	Parkinson's Disease	rs2617956	-0.511	0.213	0.016
Ability to smell	Parkinson's Disease	rs2647258	-0.501	0.213	0.019
Ability to smell	Parkinson's Disease	rs2710323	-0.491	0.214	0.021
Ability to smell	Parkinson's Disease	rs2754513	-0.479	0.213	0.025
Ability to smell	Parkinson's Disease	rs2759310	-0.480	0.212	0.024
Ability to smell	Parkinson's Disease	rs2772227	-0.472	0.211	0.025
Ability to smell	Parkinson's Disease	rs279826	-0.485	0.213	0.023
Ability to smell	Parkinson's Disease	rs2806406	-0.486	0.213	0.023
Ability to smell	Parkinson's Disease	rs28371453	-0.507	0.212	0.017
Ability to smell	Parkinson's Disease	rs28412876	-0.502	0.213	0.019
Ability to smell	Parkinson's Disease	rs28590228	-0.498	0.213	0.020
Ability to smell	Parkinson's Disease	rs2946993	-0.490	0.213	0.021
Ability to smell	Parkinson's Disease	rs2962853	-0.495	0.213	0.020
Ability to smell	Parkinson's Disease	rs2968532	-0.485	0.214	0.023
Ability to smell	Parkinson's Disease	rs303757	-0.493	0.214	0.021
Ability to smell	Parkinson's Disease	rs3087979	-0.517	0.212	0.015
Ability to smell	Parkinson's Disease	rs3099370	-0.483	0.213	0.023
Ability to smell	Parkinson's Disease	rs3124031	-0.493	0.213	0.021
Ability to smell	Parkinson's Disease	rs331992	-0.485	0.213	0.023
Ability to smell	Parkinson's Disease	rs33431	-0.496	0.213	0.020
Ability to smell	Parkinson's Disease	rs338218	-0.472	0.212	0.026
Ability to smell	Parkinson's Disease	rs34811474	-0.469	0.213	0.027

Ability to smell	Parkinson's Disease	rs35307180	-0.496	0.213	0.020
Ability to smell	Parkinson's Disease	rs35400008	-0.503	0.213	0.018
Ability to smell	Parkinson's Disease	rs35764849	-0.505	0.213	0.018
Ability to smell	Parkinson's Disease	rs358544	-0.490	0.213	0.021
Ability to smell	Parkinson's Disease	rs36092039	-0.495	0.213	0.020
Ability to smell	Parkinson's Disease	rs3756059	-0.469	0.212	0.027
Ability to smell	Parkinson's Disease	rs3764002	-0.457	0.213	0.032
Ability to smell	Parkinson's Disease	rs3784895	-0.478	0.213	0.025
Ability to smell	Parkinson's Disease	rs3802851	-0.493	0.213	0.021
Ability to smell	Parkinson's Disease	rs3803286	-0.496	0.213	0.020
Ability to smell	Parkinson's Disease	rs3808150	-0.492	0.213	0.021
Ability to smell	Parkinson's Disease	rs406388	-0.493	0.213	0.021
Ability to smell	Parkinson's Disease	rs4072917	-0.494	0.213	0.021
Ability to smell	Parkinson's Disease	rs41228	-0.504	0.213	0.018
Ability to smell	Parkinson's Disease	rs4128270	-0.493	0.213	0.021
Ability to smell	Parkinson's Disease	rs41430945	-0.495	0.213	0.020
Ability to smell	Parkinson's Disease	rs4144686	-0.495	0.213	0.020
Ability to smell	Parkinson's Disease	rs4149391	-0.484	0.213	0.023
Ability to smell	Parkinson's Disease	rs4242551	-0.508	0.213	0.017
Ability to smell	Parkinson's Disease	rs4254636	-0.501	0.213	0.018
Ability to smell	Parkinson's Disease	rs4262318	-0.487	0.213	0.022
Ability to smell	Parkinson's Disease	rs4273233	-0.494	0.213	0.021
Ability to smell	Parkinson's Disease	rs432426	-0.481	0.213	0.024
Ability to smell	Parkinson's Disease	rs4362026	-0.491	0.213	0.021
Ability to smell	Parkinson's Disease	rs4388955	-0.488	0.213	0.022
Ability to smell	Parkinson's Disease	rs4529405	-0.499	0.213	0.019
Ability to smell	Parkinson's Disease	rs4538234	-0.499	0.213	0.019
Ability to smell	Parkinson's Disease	rs454568	-0.501	0.213	0.019
Ability to smell	Parkinson's Disease	rs4565426	-0.487	0.213	0.022
Ability to smell	Parkinson's Disease	rs4567133	-0.502	0.213	0.018
Ability to smell	Parkinson's Disease	rs4577242	-0.469	0.211	0.026
Ability to smell	Parkinson's Disease	rs4639798	-0.509	0.213	0.017
Ability to smell	Parkinson's Disease	rs4656518	-0.478	0.211	0.024
Ability to smell	Parkinson's Disease	rs4702351	-0.481	0.212	0.023
Ability to smell	Parkinson's Disease	rs4742127	-0.510	0.212	0.016
Ability to smell	Parkinson's Disease	rs4777183	-0.498	0.213	0.020
Ability to smell	Parkinson's Disease	rs4778984	-0.506	0.212	0.017
Ability to smell	Parkinson's Disease	rs4799358	-0.480	0.212	0.024
Ability to smell	Parkinson's Disease	rs4805579	-0.480	0.212	0.023
Ability to smell	Parkinson's Disease	rs4902026	-0.506	0.213	0.017

Ability to smell	Parkinson's Disease	rs5343	-0.499	0.213	0.019
Ability to smell	Parkinson's Disease	rs558706	-0.503	0.213	0.018
Ability to smell	Parkinson's Disease	rs56094217	-0.497	0.213	0.020
Ability to smell	Parkinson's Disease	rs56278119	-0.492	0.213	0.021
Ability to smell	Parkinson's Disease	rs56391038	-0.501	0.213	0.019
Ability to smell	Parkinson's Disease	rs58148428	-0.489	0.213	0.022
Ability to smell	Parkinson's Disease	rs58768853	-0.506	0.213	0.017
Ability to smell	Parkinson's Disease	rs59259939	-0.498	0.214	0.020
Ability to smell	Parkinson's Disease	rs59768128	-0.498	0.213	0.019
Ability to smell	Parkinson's Disease	rs599887	-0.493	0.213	0.021
Ability to smell	Parkinson's Disease	rs6058569	-0.492	0.213	0.021
Ability to smell	Parkinson's Disease	rs60796561	-0.491	0.213	0.021
Ability to smell	Parkinson's Disease	rs61094404	-0.502	0.213	0.019
Ability to smell	Parkinson's Disease	rs62109933	-0.489	0.213	0.021
Ability to smell	Parkinson's Disease	rs62124874	-0.497	0.213	0.020
Ability to smell	Parkinson's Disease	rs62180106	-0.511	0.213	0.016
Ability to smell	Parkinson's Disease	rs62317264	-0.497	0.213	0.020
Ability to smell	Parkinson's Disease	rs62346281	-0.478	0.213	0.025
Ability to smell	Parkinson's Disease	rs62502022	-0.497	0.213	0.020
Ability to smell	Parkinson's Disease	rs6440003	-0.498	0.213	0.019
Ability to smell	Parkinson's Disease	rs6518100	-0.485	0.213	0.022
Ability to smell	Parkinson's Disease	rs6571932	-0.501	0.213	0.019
Ability to smell	Parkinson's Disease	rs6593691	-0.502	0.213	0.018
Ability to smell	Parkinson's Disease	rs6606909	-0.490	0.213	0.021
Ability to smell	Parkinson's Disease	rs6656743	-0.506	0.213	0.017
Ability to smell	Parkinson's Disease	rs6677716	-0.493	0.213	0.021
Ability to smell	Parkinson's Disease	rs6704768	-0.525	0.214	0.014
Ability to smell	Parkinson's Disease	rs6715454	-0.483	0.213	0.023
Ability to smell	Parkinson's Disease	rs6779394	-0.473	0.213	0.026
Ability to smell	Parkinson's Disease	rs68018407	-0.498	0.213	0.019
Ability to smell	Parkinson's Disease	rs6829629	-0.514	0.212	0.015
Ability to smell	Parkinson's Disease	rs687094	-0.507	0.212	0.017
Ability to smell	Parkinson's Disease	rs6873273	-0.494	0.213	0.020
Ability to smell	Parkinson's Disease	rs6943073	-0.485	0.213	0.023
Ability to smell	Parkinson's Disease	rs6956399	-0.495	0.213	0.020
Ability to smell	Parkinson's Disease	rs6971441	-0.494	0.213	0.020
Ability to smell	Parkinson's Disease	rs6973573	-0.491	0.213	0.021
Ability to smell	Parkinson's Disease	rs698769	-0.500	0.213	0.019
Ability to smell	Parkinson's Disease	rs6992179	-0.499	0.213	0.019
Ability to smell	Parkinson's Disease	rs6998822	-0.502	0.213	0.018

Ability to smell	Parkinson's Disease	rs700061	-0.489	0.213	0.022
Ability to smell	Parkinson's Disease	rs7002652	-0.480	0.212	0.024
Ability to smell	Parkinson's Disease	rs7016874	-0.513	0.212	0.015
Ability to smell	Parkinson's Disease	rs7039136	-0.491	0.213	0.021
Ability to smell	Parkinson's Disease	rs7047853	-0.487	0.213	0.022
Ability to smell	Parkinson's Disease	rs7093416	-0.492	0.213	0.021
Ability to smell	Parkinson's Disease	rs7119426	-0.501	0.213	0.019
Ability to smell	Parkinson's Disease	rs7133013	-0.502	0.213	0.018
Ability to smell	Parkinson's Disease	rs71368508	-0.494	0.213	0.020
Ability to smell	Parkinson's Disease	rs7137139	-0.493	0.213	0.021
Ability to smell	Parkinson's Disease	rs7137653	-0.497	0.213	0.020
Ability to smell	Parkinson's Disease	rs7144135	-0.540	0.217	0.013
Ability to smell	Parkinson's Disease	rs71582886	-0.501	0.213	0.019
Ability to smell	Parkinson's Disease	rs717915	-0.524	0.210	0.013
Ability to smell	Parkinson's Disease	rs7196853	-0.507	0.213	0.017
Ability to smell	Parkinson's Disease	rs7225002	-0.423	0.213	0.047
Ability to smell	Parkinson's Disease	rs72829457	-0.486	0.213	0.023
Ability to smell	Parkinson's Disease	rs73004622	-0.488	0.213	0.022
Ability to smell	Parkinson's Disease	rs73138782	-0.487	0.213	0.022
Ability to smell	Parkinson's Disease	rs7335993	-0.499	0.213	0.019
Ability to smell	Parkinson's Disease	rs73482000	-0.497	0.213	0.020
Ability to smell	Parkinson's Disease	rs7349958	-0.491	0.213	0.021
Ability to smell	Parkinson's Disease	rs7383874	-0.501	0.213	0.019
Ability to smell	Parkinson's Disease	rs7405987	-0.494	0.213	0.020
Ability to smell	Parkinson's Disease	rs7460852	-0.504	0.213	0.018
Ability to smell	Parkinson's Disease	rs7468681	-0.506	0.212	0.017
Ability to smell	Parkinson's Disease	rs75231257	-0.497	0.213	0.020
Ability to smell	Parkinson's Disease	rs7551627	-0.488	0.213	0.022
Ability to smell	Parkinson's Disease	rs7555395	-0.507	0.213	0.017
Ability to smell	Parkinson's Disease	rs7611619	-0.493	0.214	0.021
Ability to smell	Parkinson's Disease	rs76522376	-0.505	0.213	0.018
Ability to smell	Parkinson's Disease	rs7688383	-0.487	0.213	0.022
Ability to smell	Parkinson's Disease	rs77052120	-0.492	0.213	0.021
Ability to smell	Parkinson's Disease	rs7707104	-0.475	0.212	0.025
Ability to smell	Parkinson's Disease	rs7728341	-0.497	0.213	0.020
Ability to smell	Parkinson's Disease	rs773125	-0.494	0.213	0.021
Ability to smell	Parkinson's Disease	rs778138	-0.505	0.213	0.018
Ability to smell	Parkinson's Disease	rs7868227	-0.492	0.213	0.021
Ability to smell	Parkinson's Disease	rs78706681	-0.501	0.213	0.019
Ability to smell	Parkinson's Disease	rs7907344	-0.487	0.213	0.022

Ability to smell	Parkinson's Disease	rs7938634	-0.482	0.212	0.023
Ability to smell	Parkinson's Disease	rs7986422	-0.500	0.213	0.019
Ability to smell	Parkinson's Disease	rs7996330	-0.510	0.213	0.017
Ability to smell	Parkinson's Disease	rs79966207	-0.497	0.213	0.020
Ability to smell	Parkinson's Disease	rs8075338	-0.491	0.213	0.021
Ability to smell	Parkinson's Disease	rs823114	-0.572	0.206	0.006
Ability to smell	Parkinson's Disease	rs858279	-0.455	0.209	0.030
Ability to smell	Parkinson's Disease	rs858502	-0.502	0.213	0.019
Ability to smell	Parkinson's Disease	rs876493	-0.509	0.213	0.017
Ability to smell	Parkinson's Disease	rs893434	-0.500	0.213	0.019
Ability to smell	Parkinson's Disease	rs9316841	-0.492	0.213	0.021
Ability to smell	Parkinson's Disease	rs9380526	-0.507	0.213	0.017
Ability to smell	Parkinson's Disease	rs9401850	-0.489	0.213	0.021
Ability to smell	Parkinson's Disease	rs9456784	-0.486	0.213	0.022
Ability to smell	Parkinson's Disease	rs946526	-0.496	0.213	0.020
Ability to smell	Parkinson's Disease	rs9517578	-0.518	0.212	0.015
Ability to smell	Parkinson's Disease	rs9564497	-0.500	0.213	0.019
Ability to smell	Parkinson's Disease	rs9679427	-0.471	0.212	0.026
Ability to smell	Parkinson's Disease	rs9847958	-0.524	0.211	0.013
Ability to smell	Parkinson's Disease	rs9861687	-0.490	0.213	0.021
Ability to smell	Parkinson's Disease	rs9870195	-0.477	0.213	0.025
Ability to smell	Parkinson's Disease	rs9878602	-0.498	0.213	0.019
Ability to smell	Parkinson's Disease	rs9881883	-0.498	0.213	0.019
Ability to smell	Parkinson's Disease	rs9885441	-0.501	0.213	0.019
Ability to smell	Parkinson's Disease	rs9905914	-0.501	0.213	0.019
Ability to smell	Parkinson's Disease	rs9923598	-0.501	0.213	0.019
Ability to smell	Parkinson's Disease	rs992969	-0.486	0.213	0.023
Ability to smell	Parkinson's Disease	rs9935325	-0.506	0.213	0.018
Ability to smell	Parkinson's Disease	rs9975562	-0.493	0.213	0.021
Ability to smell	Parkinson's Disease	rs9992872	-0.500	0.213	0.019
Ability to smell	Parkinson's Disease	All	-0.495	0.213	0.020
Ability to smell - outliers removed	Parkinson's Disease	rs10017565	-0.528	0.205	0.010
Ability to smell - outliers removed	Parkinson's Disease	rs10041403	-0.539	0.205	0.008
Ability to smell - outliers removed	Parkinson's Disease	rs1011339	-0.551	0.205	0.007
Ability to smell - outliers removed	Parkinson's Disease	rs10143158	-0.530	0.204	0.010
Ability to smell - outliers removed	Parkinson's Disease	rs10145001	-0.545	0.205	0.008
Ability to smell - outliers removed	Parkinson's Disease	rs10169534	-0.560	0.203	0.006
Ability to smell - outliers removed	Parkinson's Disease	rs10175449	-0.546	0.205	0.008
Ability to smell - outliers removed	Parkinson's Disease	rs1018466	-0.547	0.205	0.007
Ability to smell - outliers removed	Parkinson's Disease	rs10189857	-0.522	0.205	0.011

Ability to smell - outliers removed	Parkinson's Disease	rs10228058	-0.525	0.204	0.010
Ability to smell - outliers removed	Parkinson's Disease	rs10408179	-0.517	0.204	0.011
Ability to smell - outliers removed	Parkinson's Disease	rs10498754	-0.540	0.205	0.008
Ability to smell - outliers removed	Parkinson's Disease	rs10516927	-0.546	0.205	0.008
Ability to smell - outliers removed	Parkinson's Disease	rs1051695	-0.539	0.205	0.009
Ability to smell - outliers removed	Parkinson's Disease	rs1060402	-0.547	0.205	0.007
Ability to smell - outliers removed	Parkinson's Disease	rs1065	-0.529	0.205	0.010
Ability to smell - outliers removed	Parkinson's Disease	rs10801274	-0.540	0.205	0.008
Ability to smell - outliers removed	Parkinson's Disease	rs10840683	-0.552	0.204	0.007
Ability to smell - outliers removed	Parkinson's Disease	rs10865701	-0.534	0.205	0.009
Ability to smell - outliers removed	Parkinson's Disease	rs11049380	-0.541	0.205	0.008
Ability to smell - outliers removed	Parkinson's Disease	rs11112945	-0.545	0.205	0.008
Ability to smell - outliers removed	Parkinson's Disease	rs11125047	-0.544	0.205	0.008
Ability to smell - outliers removed	Parkinson's Disease	rs111300896	-0.541	0.205	0.008
Ability to smell - outliers removed	Parkinson's Disease	rs11162408	-0.515	0.203	0.011
Ability to smell - outliers removed	Parkinson's Disease	rs11204752	-0.580	0.204	0.004
Ability to smell - outliers removed	Parkinson's Disease	rs11246795	-0.542	0.205	0.008
Ability to smell - outliers removed	Parkinson's Disease	rs113408217	-0.536	0.205	0.009
Ability to smell - outliers removed	Parkinson's Disease	rs1138120	-0.537	0.206	0.009
Ability to smell - outliers removed	Parkinson's Disease	rs113931022	-0.527	0.205	0.010
Ability to smell - outliers removed	Parkinson's Disease	rs11545169	-0.566	0.204	0.006
Ability to smell - outliers removed	Parkinson's Disease	rs1158570	-0.524	0.204	0.010
Ability to smell - outliers removed	Parkinson's Disease	rs11606078	-0.533	0.205	0.009
Ability to smell - outliers removed	Parkinson's Disease	rs11641598	-0.533	0.205	0.009
Ability to smell - outliers removed	Parkinson's Disease	rs11647633	-0.535	0.205	0.009
Ability to smell - outliers removed	Parkinson's Disease	rs1167264	-0.535	0.205	0.009
Ability to smell - outliers removed	Parkinson's Disease	rs11682175	-0.527	0.205	0.010
Ability to smell - outliers removed	Parkinson's Disease	rs11736548	-0.544	0.205	0.008
Ability to smell - outliers removed	Parkinson's Disease	rs11737962	-0.532	0.205	0.009
Ability to smell - outliers removed	Parkinson's Disease	rs11743259	-0.524	0.205	0.011
Ability to smell - outliers removed	Parkinson's Disease	rs11787216	-0.541	0.205	0.008
Ability to smell - outliers removed	Parkinson's Disease	rs118093058	-0.526	0.204	0.010
Ability to smell - outliers removed	Parkinson's Disease	rs11816241	-0.534	0.205	0.009
Ability to smell - outliers removed	Parkinson's Disease	rs1198598	-0.522	0.204	0.010
Ability to smell - outliers removed	Parkinson's Disease	rs12031155	-0.547	0.205	0.008
Ability to smell - outliers removed	Parkinson's Disease	rs1203850	-0.543	0.205	0.008
Ability to smell - outliers removed	Parkinson's Disease	rs12055438	-0.543	0.205	0.008
Ability to smell - outliers removed	Parkinson's Disease	rs12120481	-0.549	0.205	0.007
Ability to smell - outliers removed	Parkinson's Disease	rs12139295	-0.552	0.205	0.007
Ability to smell - outliers removed	Parkinson's Disease	rs12149498	-0.554	0.205	0.007

Ability to smell - outliers removed	Parkinson's Disease	rs12154492	-0.539	0.205	0.009
Ability to smell - outliers removed	Parkinson's Disease	rs12160976	-0.532	0.205	0.009
Ability to smell - outliers removed	Parkinson's Disease	rs1217353	-0.538	0.205	0.009
Ability to smell - outliers removed	Parkinson's Disease	rs12457322	-0.526	0.204	0.010
Ability to smell - outliers removed	Parkinson's Disease	rs12544554	-0.537	0.205	0.009
Ability to smell - outliers removed	Parkinson's Disease	rs12600322	-0.545	0.205	0.008
Ability to smell - outliers removed	Parkinson's Disease	rs1265565	-0.540	0.205	0.009
Ability to smell - outliers removed	Parkinson's Disease	rs12665528	-0.532	0.205	0.009
Ability to smell - outliers removed	Parkinson's Disease	rs12808129	-0.541	0.205	0.008
Ability to smell - outliers removed	Parkinson's Disease	rs12823540	-0.546	0.205	0.008
Ability to smell - outliers removed	Parkinson's Disease	rs1283200	-0.539	0.205	0.009
Ability to smell - outliers removed	Parkinson's Disease	rs12917189	-0.567	0.203	0.005
Ability to smell - outliers removed	Parkinson's Disease	rs12924872	-0.559	0.205	0.006
Ability to smell - outliers removed	Parkinson's Disease	rs13030947	-0.538	0.205	0.009
Ability to smell - outliers removed	Parkinson's Disease	rs13060816	-0.544	0.205	0.008
Ability to smell - outliers removed	Parkinson's Disease	rs13166639	-0.535	0.205	0.009
Ability to smell - outliers removed	Parkinson's Disease	rs13197257	-0.545	0.205	0.008
Ability to smell - outliers removed	Parkinson's Disease	rs13214277	-0.546	0.205	0.008
Ability to smell - outliers removed	Parkinson's Disease	rs13226650	-0.553	0.205	0.007
Ability to smell - outliers removed	Parkinson's Disease	rs13239186	-0.565	0.204	0.006
Ability to smell - outliers removed	Parkinson's Disease	rs13246732	-0.542	0.205	0.008
Ability to smell - outliers removed	Parkinson's Disease	rs13269741	-0.556	0.205	0.007
Ability to smell - outliers removed	Parkinson's Disease	rs13389333	-0.530	0.204	0.010
Ability to smell - outliers removed	Parkinson's Disease	rs133908	-0.547	0.205	0.007
Ability to smell - outliers removed	Parkinson's Disease	rs1342929	-0.546	0.205	0.008
Ability to smell - outliers removed	Parkinson's Disease	rs1366024	-0.572	0.203	0.005
Ability to smell - outliers removed	Parkinson's Disease	rs1393065	-0.537	0.205	0.009
Ability to smell - outliers removed	Parkinson's Disease	rs1425548	-0.522	0.204	0.011
Ability to smell - outliers removed	Parkinson's Disease	rs144183806	-0.535	0.205	0.009
Ability to smell - outliers removed	Parkinson's Disease	rs1466234	-0.544	0.205	0.008
Ability to smell - outliers removed	Parkinson's Disease	rs1532729	-0.532	0.205	0.009
Ability to smell - outliers removed	Parkinson's Disease	rs1544861	-0.530	0.205	0.010
Ability to smell - outliers removed	Parkinson's Disease	rs1606182	-0.537	0.205	0.009
Ability to smell - outliers removed	Parkinson's Disease	rs1623177	-0.547	0.205	0.008
Ability to smell - outliers removed	Parkinson's Disease	rs1638526	-0.547	0.205	0.008
Ability to smell - outliers removed	Parkinson's Disease	rs16835705	-0.556	0.205	0.007
Ability to smell - outliers removed	Parkinson's Disease	rs1693493	-0.525	0.204	0.010
Ability to smell - outliers removed	Parkinson's Disease	rs1712957	-0.552	0.205	0.007
Ability to smell - outliers removed	Parkinson's Disease	rs17153017	-0.550	0.205	0.007
Ability to smell - outliers removed	Parkinson's Disease	rs17283010	-0.483	0.203	0.017

Ability to smell - outliers removed	Parkinson's Disease	rs17314727	-0.562	0.205	0.006
Ability to smell - outliers removed	Parkinson's Disease	rs17648524	-0.533	0.205	0.009
Ability to smell - outliers removed	Parkinson's Disease	rs17669337	-0.549	0.205	0.007
Ability to smell - outliers removed	Parkinson's Disease	rs17766830	-0.531	0.205	0.009
Ability to smell - outliers removed	Parkinson's Disease	rs17767336	-0.548	0.204	0.007
Ability to smell - outliers removed	Parkinson's Disease	rs17776374	-0.538	0.205	0.009
Ability to smell - outliers removed	Parkinson's Disease	rs17801787	-0.530	0.204	0.009
Ability to smell - outliers removed	Parkinson's Disease	rs1796974	-0.533	0.205	0.009
Ability to smell - outliers removed	Parkinson's Disease	rs1921622	-0.559	0.204	0.006
Ability to smell - outliers removed	Parkinson's Disease	rs1933720	-0.530	0.205	0.010
Ability to smell - outliers removed	Parkinson's Disease	rs2047812	-0.541	0.205	0.008
Ability to smell - outliers removed	Parkinson's Disease	rs2071164	-0.533	0.205	0.009
Ability to smell - outliers removed	Parkinson's Disease	rs2105619	-0.536	0.205	0.009
Ability to smell - outliers removed	Parkinson's Disease	rs2130519	-0.539	0.205	0.008
Ability to smell - outliers removed	Parkinson's Disease	rs2155077	-0.542	0.205	0.008
Ability to smell - outliers removed	Parkinson's Disease	rs2170719	-0.543	0.205	0.008
Ability to smell - outliers removed	Parkinson's Disease	rs2192564	-0.548	0.205	0.007
Ability to smell - outliers removed	Parkinson's Disease	rs2198234	-0.527	0.206	0.010
Ability to smell - outliers removed	Parkinson's Disease	rs2243504	-0.546	0.205	0.008
Ability to smell - outliers removed	Parkinson's Disease	rs2246585	-0.535	0.205	0.009
Ability to smell - outliers removed	Parkinson's Disease	rs2287431	-0.535	0.205	0.009
Ability to smell - outliers removed	Parkinson's Disease	rs2301579	-0.547	0.205	0.008
Ability to smell - outliers removed	Parkinson's Disease	rs2305191	-0.514	0.204	0.012
Ability to smell - outliers removed	Parkinson's Disease	rs2332058	-0.544	0.205	0.008
Ability to smell - outliers removed	Parkinson's Disease	rs2360635	-0.552	0.204	0.007
Ability to smell - outliers removed	Parkinson's Disease	rs2361050	-0.516	0.204	0.011
Ability to smell - outliers removed	Parkinson's Disease	rs2374577	-0.533	0.205	0.009
Ability to smell - outliers removed	Parkinson's Disease	rs2467997	-0.543	0.205	0.008
Ability to smell - outliers removed	Parkinson's Disease	rs249623	-0.536	0.205	0.009
Ability to smell - outliers removed	Parkinson's Disease	rs2510652	-0.535	0.205	0.009
Ability to smell - outliers removed	Parkinson's Disease	rs2593163	-0.546	0.205	0.008
Ability to smell - outliers removed	Parkinson's Disease	rs2612607	-0.547	0.205	0.008
Ability to smell - outliers removed	Parkinson's Disease	rs2614893	-0.537	0.205	0.009
Ability to smell - outliers removed	Parkinson's Disease	rs2617956	-0.558	0.204	0.006
Ability to smell - outliers removed	Parkinson's Disease	rs2710323	-0.537	0.205	0.009
Ability to smell - outliers removed	Parkinson's Disease	rs2754513	-0.523	0.205	0.011
Ability to smell - outliers removed	Parkinson's Disease	rs2759310	-0.524	0.204	0.010
Ability to smell - outliers removed	Parkinson's Disease	rs2772227	-0.516	0.203	0.011
Ability to smell - outliers removed	Parkinson's Disease	rs279826	-0.530	0.205	0.010
Ability to smell - outliers removed	Parkinson's Disease	rs2806406	-0.531	0.205	0.010

Ability to smell - outliers removed	Parkinson's Disease	rs28371453	-0.554	0.204	0.007
Ability to smell - outliers removed	Parkinson's Disease	rs28412876	-0.548	0.205	0.008
Ability to smell - outliers removed	Parkinson's Disease	rs28590228	-0.543	0.205	0.008
Ability to smell - outliers removed	Parkinson's Disease	rs28780110	-0.538	0.205	0.009
Ability to smell - outliers removed	Parkinson's Disease	rs2946993	-0.535	0.205	0.009
Ability to smell - outliers removed	Parkinson's Disease	rs2968532	-0.530	0.206	0.010
Ability to smell - outliers removed	Parkinson's Disease	rs303757	-0.539	0.206	0.009
Ability to smell - outliers removed	Parkinson's Disease	rs3087979	-0.564	0.203	0.006
Ability to smell - outliers removed	Parkinson's Disease	rs3099370	-0.528	0.204	0.010
Ability to smell - outliers removed	Parkinson's Disease	rs331992	-0.530	0.205	0.010
Ability to smell - outliers removed	Parkinson's Disease	rs33431	-0.541	0.205	0.008
Ability to smell - outliers removed	Parkinson's Disease	rs338218	-0.516	0.203	0.011
Ability to smell - outliers removed	Parkinson's Disease	rs34811474	-0.513	0.204	0.012
Ability to smell - outliers removed	Parkinson's Disease	rs35764849	-0.551	0.205	0.007
Ability to smell - outliers removed	Parkinson's Disease	rs36092039	-0.541	0.205	0.008
Ability to smell - outliers removed	Parkinson's Disease	rs3756059	-0.513	0.204	0.012
Ability to smell - outliers removed	Parkinson's Disease	rs3764002	-0.500	0.205	0.014
Ability to smell - outliers removed	Parkinson's Disease	rs3784895	-0.522	0.204	0.011
Ability to smell - outliers removed	Parkinson's Disease	rs3802851	-0.538	0.205	0.009
Ability to smell - outliers removed	Parkinson's Disease	rs3803286	-0.541	0.205	0.008
Ability to smell - outliers removed	Parkinson's Disease	rs3998158	-0.560	0.205	0.006
Ability to smell - outliers removed	Parkinson's Disease	rs406388	-0.539	0.205	0.009
Ability to smell - outliers removed	Parkinson's Disease	rs4072917	-0.539	0.205	0.009
Ability to smell - outliers removed	Parkinson's Disease	rs41228	-0.550	0.205	0.007
Ability to smell - outliers removed	Parkinson's Disease	rs4128270	-0.538	0.205	0.009
Ability to smell - outliers removed	Parkinson's Disease	rs4144686	-0.540	0.205	0.008
Ability to smell - outliers removed	Parkinson's Disease	rs4149391	-0.529	0.205	0.010
Ability to smell - outliers removed	Parkinson's Disease	rs4242551	-0.554	0.204	0.007
Ability to smell - outliers removed	Parkinson's Disease	rs4254636	-0.547	0.204	0.007
Ability to smell - outliers removed	Parkinson's Disease	rs4262318	-0.532	0.205	0.009
Ability to smell - outliers removed	Parkinson's Disease	rs4273233	-0.540	0.205	0.009
Ability to smell - outliers removed	Parkinson's Disease	rs432426	-0.526	0.205	0.010
Ability to smell - outliers removed	Parkinson's Disease	rs4362026	-0.536	0.205	0.009
Ability to smell - outliers removed	Parkinson's Disease	rs4388955	-0.533	0.205	0.009
Ability to smell - outliers removed	Parkinson's Disease	rs4529405	-0.544	0.205	0.008
Ability to smell - outliers removed	Parkinson's Disease	rs4538234	-0.545	0.205	0.008
Ability to smell - outliers removed	Parkinson's Disease	rs454568	-0.547	0.205	0.008
Ability to smell - outliers removed	Parkinson's Disease	rs4565426	-0.532	0.205	0.009
Ability to smell - outliers removed	Parkinson's Disease	rs4567133	-0.548	0.205	0.007
Ability to smell - outliers removed	Parkinson's Disease	rs4639798	-0.556	0.205	0.007

Ability to smell - outliers removed	Parkinson's Disease	rs4702351	-0.526	0.204	0.010
Ability to smell - outliers removed	Parkinson's Disease	rs4742127	-0.557	0.204	0.006
Ability to smell - outliers removed	Parkinson's Disease	rs4777183	-0.543	0.205	0.008
Ability to smell - outliers removed	Parkinson's Disease	rs4799358	-0.524	0.204	0.010
Ability to smell - outliers removed	Parkinson's Disease	rs4805579	-0.525	0.203	0.010
Ability to smell - outliers removed	Parkinson's Disease	rs4902026	-0.552	0.205	0.007
Ability to smell - outliers removed	Parkinson's Disease	rs5343	-0.545	0.205	0.008
Ability to smell - outliers removed	Parkinson's Disease	rs558706	-0.549	0.205	0.007
Ability to smell - outliers removed	Parkinson's Disease	rs56094217	-0.542	0.205	0.008
Ability to smell - outliers removed	Parkinson's Disease	rs56391038	-0.547	0.205	0.008
Ability to smell - outliers removed	Parkinson's Disease	rs58148428	-0.534	0.205	0.009
Ability to smell - outliers removed	Parkinson's Disease	rs58768853	-0.553	0.205	0.007
Ability to smell - outliers removed	Parkinson's Disease	rs59259939	-0.544	0.206	0.008
Ability to smell - outliers removed	Parkinson's Disease	rs599887	-0.539	0.205	0.009
Ability to smell - outliers removed	Parkinson's Disease	rs6058569	-0.538	0.205	0.009
Ability to smell - outliers removed	Parkinson's Disease	rs60796561	-0.536	0.205	0.009
Ability to smell - outliers removed	Parkinson's Disease	rs61094404	-0.548	0.205	0.008
Ability to smell - outliers removed	Parkinson's Disease	rs62109933	-0.534	0.205	0.009
Ability to smell - outliers removed	Parkinson's Disease	rs62124874	-0.543	0.205	0.008
Ability to smell - outliers removed	Parkinson's Disease	rs62180106	-0.558	0.204	0.006
Ability to smell - outliers removed	Parkinson's Disease	rs62317264	-0.543	0.205	0.008
Ability to smell - outliers removed	Parkinson's Disease	rs62346281	-0.523	0.205	0.011
Ability to smell - outliers removed	Parkinson's Disease	rs62502022	-0.542	0.205	0.008
Ability to smell - outliers removed	Parkinson's Disease	rs6440003	-0.544	0.205	0.008
Ability to smell - outliers removed	Parkinson's Disease	rs6518100	-0.530	0.204	0.010
Ability to smell - outliers removed	Parkinson's Disease	rs6571932	-0.547	0.205	0.008
Ability to smell - outliers removed	Parkinson's Disease	rs6606909	-0.535	0.205	0.009
Ability to smell - outliers removed	Parkinson's Disease	rs6656743	-0.553	0.205	0.007
Ability to smell - outliers removed	Parkinson's Disease	rs6677716	-0.538	0.205	0.009
Ability to smell - outliers removed	Parkinson's Disease	rs6704768	-0.573	0.206	0.005
Ability to smell - outliers removed	Parkinson's Disease	rs6715454	-0.528	0.204	0.010
Ability to smell - outliers removed	Parkinson's Disease	rs6779394	-0.518	0.205	0.011
Ability to smell - outliers removed	Parkinson's Disease	rs68018407	-0.544	0.205	0.008
Ability to smell - outliers removed	Parkinson's Disease	rs6829629	-0.561	0.204	0.006
Ability to smell - outliers removed	Parkinson's Disease	rs687094	-0.553	0.204	0.007
Ability to smell - outliers removed	Parkinson's Disease	rs6873273	-0.539	0.205	0.009
Ability to smell - outliers removed	Parkinson's Disease	rs6943073	-0.530	0.205	0.010
Ability to smell - outliers removed	Parkinson's Disease	rs6956399	-0.540	0.205	0.008
Ability to smell - outliers removed	Parkinson's Disease	rs6971441	-0.540	0.205	0.008
Ability to smell - outliers removed	Parkinson's Disease	rs698769	-0.546	0.205	0.008

Ability to smell - outliers removed	Parkinson's Disease	rs6992179	-0.544	0.205	0.008
Ability to smell - outliers removed	Parkinson's Disease	rs6998822	-0.548	0.205	0.007
Ability to smell - outliers removed	Parkinson's Disease	rs700061	-0.534	0.205	0.009
Ability to smell - outliers removed	Parkinson's Disease	rs7002652	-0.525	0.204	0.010
Ability to smell - outliers removed	Parkinson's Disease	rs7016874	-0.560	0.204	0.006
Ability to smell - outliers removed	Parkinson's Disease	rs7039136	-0.536	0.205	0.009
Ability to smell - outliers removed	Parkinson's Disease	rs7047853	-0.532	0.204	0.009
Ability to smell - outliers removed	Parkinson's Disease	rs7119426	-0.547	0.205	0.008
Ability to smell - outliers removed	Parkinson's Disease	rs7133013	-0.548	0.205	0.007
Ability to smell - outliers removed	Parkinson's Disease	rs71368508	-0.540	0.205	0.008
Ability to smell - outliers removed	Parkinson's Disease	rs7137139	-0.538	0.205	0.009
Ability to smell - outliers removed	Parkinson's Disease	rs7137653	-0.543	0.205	0.008
Ability to smell - outliers removed	Parkinson's Disease	rs7144135	-0.590	0.208	0.005
Ability to smell - outliers removed	Parkinson's Disease	rs71582886	-0.547	0.205	0.008
Ability to smell - outliers removed	Parkinson's Disease	rs717915	-0.571	0.201	0.005
Ability to smell - outliers removed	Parkinson's Disease	rs7196853	-0.553	0.205	0.007
Ability to smell - outliers removed	Parkinson's Disease	rs7225002	-0.464	0.204	0.023
Ability to smell - outliers removed	Parkinson's Disease	rs72829457	-0.531	0.205	0.010
Ability to smell - outliers removed	Parkinson's Disease	rs73004622	-0.533	0.205	0.009
Ability to smell - outliers removed	Parkinson's Disease	rs73138782	-0.532	0.205	0.009
Ability to smell - outliers removed	Parkinson's Disease	rs7335993	-0.544	0.205	0.008
Ability to smell - outliers removed	Parkinson's Disease	rs73482000	-0.543	0.205	0.008
Ability to smell - outliers removed	Parkinson's Disease	rs7349958	-0.537	0.205	0.009
Ability to smell - outliers removed	Parkinson's Disease	rs7383874	-0.547	0.205	0.008
Ability to smell - outliers removed	Parkinson's Disease	rs7405987	-0.540	0.205	0.008
Ability to smell - outliers removed	Parkinson's Disease	rs7460852	-0.550	0.205	0.007
Ability to smell - outliers removed	Parkinson's Disease	rs7468681	-0.552	0.204	0.007
Ability to smell - outliers removed	Parkinson's Disease	rs75231257	-0.543	0.205	0.008
Ability to smell - outliers removed	Parkinson's Disease	rs7551627	-0.533	0.205	0.009
Ability to smell - outliers removed	Parkinson's Disease	rs7555395	-0.553	0.205	0.007
Ability to smell - outliers removed	Parkinson's Disease	rs7611619	-0.539	0.206	0.009
Ability to smell - outliers removed	Parkinson's Disease	rs76522376	-0.551	0.204	0.007
Ability to smell - outliers removed	Parkinson's Disease	rs7688383	-0.532	0.205	0.009
Ability to smell - outliers removed	Parkinson's Disease	rs77052120	-0.537	0.205	0.009
Ability to smell - outliers removed	Parkinson's Disease	rs7707104	-0.520	0.204	0.011
Ability to smell - outliers removed	Parkinson's Disease	rs7728341	-0.543	0.205	0.008
Ability to smell - outliers removed	Parkinson's Disease	rs773125	-0.539	0.205	0.009
Ability to smell - outliers removed	Parkinson's Disease	rs778138	-0.551	0.205	0.007
Ability to smell - outliers removed	Parkinson's Disease	rs7868227	-0.537	0.205	0.009
Ability to smell - outliers removed	Parkinson's Disease	rs78706681	-0.546	0.205	0.008

Ability to smell - outliers removed	Parkinson's Disease	rs7907344	-0.532	0.205	0.009
Ability to smell - outliers removed	Parkinson's Disease	rs7986422	-0.546	0.205	0.008
Ability to smell - outliers removed	Parkinson's Disease	rs7996330	-0.557	0.205	0.007
Ability to smell - outliers removed	Parkinson's Disease	rs79966207	-0.542	0.205	0.008
Ability to smell - outliers removed	Parkinson's Disease	rs8075338	-0.536	0.205	0.009
Ability to smell - outliers removed	Parkinson's Disease	rs858502	-0.548	0.205	0.008
Ability to smell - outliers removed	Parkinson's Disease	rs876493	-0.555	0.205	0.007
Ability to smell - outliers removed	Parkinson's Disease	rs9316841	-0.538	0.205	0.009
Ability to smell - outliers removed	Parkinson's Disease	rs9456784	-0.530	0.204	0.009
Ability to smell - outliers removed	Parkinson's Disease	rs946526	-0.541	0.205	0.008
Ability to smell - outliers removed	Parkinson's Disease	rs9517578	-0.565	0.203	0.005
Ability to smell - outliers removed	Parkinson's Disease	rs9564497	-0.545	0.205	0.008
Ability to smell - outliers removed	Parkinson's Disease	rs9679427	-0.515	0.203	0.011
Ability to smell - outliers removed	Parkinson's Disease	rs9847958	-0.571	0.203	0.005
Ability to smell - outliers removed	Parkinson's Disease	rs9861687	-0.535	0.205	0.009
Ability to smell - outliers removed	Parkinson's Disease	rs9870195	-0.522	0.204	0.011
Ability to smell - outliers removed	Parkinson's Disease	rs9878602	-0.544	0.205	0.008
Ability to smell - outliers removed	Parkinson's Disease	rs9881883	-0.544	0.205	0.008
Ability to smell - outliers removed	Parkinson's Disease	rs9885441	-0.547	0.205	0.008
Ability to smell - outliers removed	Parkinson's Disease	rs9905914	-0.547	0.205	0.008
Ability to smell - outliers removed	Parkinson's Disease	rs9923598	-0.547	0.205	0.008
Ability to smell - outliers removed	Parkinson's Disease	rs992969	-0.531	0.205	0.010
Ability to smell - outliers removed	Parkinson's Disease	rs9935325	-0.552	0.205	0.007
Ability to smell - outliers removed	Parkinson's Disease	rs9975562	-0.538	0.205	0.009
Ability to smell - outliers removed	Parkinson's Disease	rs9992872	-0.546	0.205	0.008
Ability to smell - outliers removed	Parkinson's Disease	All	-0.540	0.204	0.008

Supplementary Table 5 – Variants used for MR and Single Variant MR results (Wald Ratio). CHR: chromosome number; BP: basepair position (hg19); BETA: effect size in logOR scale; SE: standard error of effect size; P: *p*-value; Removed by MR-PRESSO: the variant was removed by MR-PRESSO distortion test.

					Exposure			Outcome			Wald Ratio			
**Exposure**	**Outcome**	**SNP**	**CHR**	**BP**	**BETA**	**SE**	**P**	**BETA**	**SE**	**P**	**BETA**	**SE**	**P**	**Removed by MR-PRESSO**
Parkinson's Disease	Ability to smell	rs35749011	1	155135036	0.698	0.081	4.71E-18	-0.020	0.005	4.83E-05	-0.028	0.007	4.83E-05	FALSE
Parkinson's Disease	Ability to smell	rs2153904	1	205642790	0.157	0.026	1.99E-09	0.002	0.001	0.097	0.016	0.009	0.097	TRUE
Parkinson's Disease	Ability to smell	rs4613239	2	169119609	0.181	0.028	5.96E-11	-0.006	0.002	4.14E-04	-0.032	0.009	4.14E-04	FALSE
Parkinson's Disease	Ability to smell	rs12637471	3	182762437	-0.166	0.024	9.78E-12	4.77E-04	0.001	0.730	-0.003	0.008	0.730	FALSE
Parkinson's Disease	Ability to smell	rs34311866	4	951947	0.219	0.027	1.54E-16	-0.001	0.001	0.528	-0.004	0.006	0.528	TRUE
Parkinson's Disease	Ability to smell	rs4698412	4	15737348	-0.131	0.019	2.21E-12	0.004	0.001	0.001	-0.028	0.008	0.001	FALSE
Parkinson's Disease	Ability to smell	rs356182	4	90626111	0.262	0.022	4.93E-34	-1.52E-04	0.001	0.896	-0.001	0.004	0.896	TRUE
Parkinson's Disease	Ability to smell	rs75646569	5	60345424	0.184	0.030	4.39E-10	-0.003	0.002	0.115	-0.015	0.010	0.115	FALSE
Parkinson's Disease	Ability to smell	rs2263418	12	40582993	0.167	0.029	1.43E-08	-0.004	0.002	0.046	-0.021	0.011	0.046	FALSE
Parkinson's Disease	Ability to smell	rs10847864	12	123326598	0.132	0.020	3.78E-11	-0.002	0.001	0.041	-0.018	0.009	0.041	FALSE
Parkinson's Disease	Ability to smell	rs4889603	16	30982225	0.115	0.021	2.90E-08	-0.004	0.001	1.10E-04	-0.037	0.010	1.10E-04	FALSE
Parkinson's Disease	Ability to smell	rs199452	17	44801340	-0.179	0.026	6.46E-12	0.020	0.001	2.05E-53	-0.110	0.007	2.03E-53	TRUE
Parkinson's Disease	Ability to smell	rs4588066	18	40672964	0.115	0.020	6.86E-09	-0.003	0.001	0.018	-0.024	0.010	0.018	FALSE
Ability to smell	Parkinson's Disease	rs1393065	1	3099105	0.008	0.001	3.52E-12	-0.014	0.030	0.632	-1.685	3.512	0.631	FALSE
Ability to smell	Parkinson's Disease	rs599887	1	38240300	-0.007	0.001	3.35E-11	0.006	0.020	0.761	-0.836	2.754	0.762	FALSE
Ability to smell	Parkinson's Disease	rs946526	1	46487168	-0.018	0.003	1.67E-10	0.004	0.056	0.942	-0.230	3.107	0.941	FALSE
Ability to smell	Parkinson's Disease	rs1167264	1	49933045	-0.010	0.001	1.57E-13	0.024	0.034	0.478	-2.441	3.428	0.476	FALSE
Ability to smell	Parkinson's Disease	rs12031155	1	53714139	-0.009	0.001	1.08E-16	-0.004	0.019	0.854	0.379	2.056	0.854	FALSE
Ability to smell	Parkinson's Disease	rs2806406	1	58538738	0.007	0.001	7.57E-10	-0.029	0.024	0.222	-4.253	3.484	0.222	FALSE
Ability to smell	Parkinson's Disease	rs1009368	1	58724719	0.007	0.002	4.04E-06	0.035	0.041	0.398	4.701	5.569	0.399	FALSE

Ability to smell	Parkinson's Disease	rs1796974	1	70174710	-0.008	0.001	1.10E-13	0.025	0.027	0.367	-3.006	3.321	0.365	FALSE
Ability to smell	Parkinson's Disease	rs7551627	1	72960531	-0.009	0.001	4.55E-12	0.020	0.024	0.411	-2.134	2.592	0.410	FALSE
Ability to smell	Parkinson's Disease	rs11162408	1	78495175	-0.009	0.001	3.26E-10	0.065	0.026	0.011	-7.654	2.998	0.011	FALSE
Ability to smell	Parkinson's Disease	rs4362026	1	81890814	-0.007	0.001	2.56E-11	0.017	0.028	0.528	-2.364	3.758	0.529	FALSE
Ability to smell	Parkinson's Disease	rs7555395	1	93610935	0.009	0.001	1.23E-11	0.022	0.023	0.348	2.533	2.695	0.347	FALSE
Ability to smell	Parkinson's Disease	rs6593691	1	96412885	-0.006	0.001	9.33E-07	-0.037	0.032	0.258	5.692	5.024	0.257	FALSE
Ability to smell	Parkinson's Disease	rs1198598	1	98535375	0.008	0.001	1.11E-09	-0.060	0.028	0.033	-7.164	3.368	0.033	FALSE
Ability to smell	Parkinson's Disease	rs2360635	1	110604747	0.007	0.001	8.81E-10	0.040	0.028	0.160	5.551	3.953	0.160	FALSE
Ability to smell	Parkinson's Disease	rs77052120	1	110765398	0.013	0.002	1.34E-11	-0.015	0.031	0.638	-1.160	2.471	0.639	FALSE
Ability to smell	Parkinson's Disease	rs3124031	1	112843384	0.006	0.001	5.72E-08	-0.010	0.025	0.674	-1.607	3.822	0.674	FALSE
Ability to smell	Parkinson's Disease	rs12144309	1	114315493	-0.007	0.001	2.56E-06	-0.017	0.028	0.535	2.657	4.285	0.535	FALSE
Ability to smell	Parkinson's Disease	rs11204752	1	150958133	0.010	0.001	1.59E-18	0.043	0.019	0.026	4.446	1.990	0.025	FALSE
Ability to smell	Parkinson's Disease	rs1342929	1	151449138	0.009	0.001	8.27E-11	0.008	0.025	0.735	0.912	2.714	0.737	FALSE
Ability to smell	Parkinson's Disease	rs6656743	1	154553450	0.007	0.001	1.81E-09	0.021	0.021	0.320	2.883	2.911	0.322	FALSE
Ability to smell	Parkinson's Disease	rs12120481	1	166683359	-0.011	0.002	5.48E-11	-0.026	0.034	0.440	2.479	3.215	0.441	FALSE
Ability to smell	Parkinson's Disease	rs4656518	1	166968972	0.005	0.001	4.83E-05	-0.068	0.023	0.003	-14.869	4.963	0.003	FALSE
Ability to smell	Parkinson's Disease	rs2332058	1	181046911	-0.007	0.001	6.94E-10	-0.012	0.028	6.60E-01	1.787	4.069	6.60E-01	
Ability to smell	Parkinson's Disease	rs10801274	1	189176930	0.007	0.001	1.10E-11	-0.004	0.023	0.869	-0.511	3.095	0.869	FALSE
Ability to smell	Parkinson's Disease	rs6677716	1	194380106	-0.010	0.002	2.99E-08	0.023	0.049	0.641	-2.296	4.922	0.641	FALSE
Ability to smell	Parkinson's Disease	rs2772227	1	204615400	0.007	0.001	2.25E-08	-0.069	0.024	0.004	-10.469	3.605	0.004	FALSE
Ability to smell	Parkinson's Disease	rs12139295	1	205186873	0.011	0.001	1.18E-14	0.036	0.035	0.293	3.325	3.161	0.293	FALSE
Ability to smell	Parkinson's Disease	rs823114	1	205719532	-0.009	0.001	4.42E-15	-0.105	0.019	0.000	12.168	2.202	0.000	FALSE
Ability to smell	Parkinson's Disease	rs1244995	1	215025646	0.006	0.001	3.06E-06	-0.031	0.029	0.298	-5.492	5.277	0.298	FALSE
Ability to smell	Parkinson's Disease	rs16835705	1	237908911	-0.009	0.001	4.18E-13	-0.031	0.025	0.210	3.550	2.833	0.210	FALSE
Ability to smell	Parkinson's Disease	rs4639798	1	248515703	0.008	0.001	3.10E-12	0.020	0.020	0.315	2.493	2.481	0.315	FALSE
Ability to smell	Parkinson's Disease	rs10175449	2	8023091	0.006	0.001	1.90E-08	0.019	0.028	0.499	2.978	4.412	0.500	FALSE
Ability to smell	Parkinson's Disease	rs62124874	2	22655354	-0.010	0.002	2.19E-08	-0.010	0.043	0.825	0.974	4.378	0.824	FALSE

Ability to smell	Parkinson's Disease	rs2754513	2	32782763	0.008	0.001	2.80E-13	-0.028	0.019	0.141	-3.412	2.318	0.141	FALSE
Ability to smell	Parkinson's Disease	rs698769	2	44579471	-0.015	0.002	4.34E-11	-0.035	0.057	0.540	2.346	3.826	0.540	FALSE
Ability to smell	Parkinson's Disease	rs11125047	2	46310381	-0.008	0.001	1.10E-11	-0.011	0.030	0.706	1.397	3.714	0.707	FALSE
Ability to smell	Parkinson's Disease	rs12988253	2	49503873	-0.006	0.001	9.54E-08	0.029	0.030	0.346	-4.417	4.681	0.345	FALSE
Ability to smell	Parkinson's Disease	rs11682175	2	57987593	-0.008	0.001	1.78E-12	0.026	0.020	0.199	-3.369	2.630	0.200	FALSE
Ability to smell	Parkinson's Disease	rs2192564	2	59472899	-0.010	0.002	8.22E-10	-0.035	0.040	0.376	3.681	4.162	0.376	FALSE
Ability to smell	Parkinson's Disease	rs10189857	2	60713235	0.009	0.001	5.85E-16	-0.029	0.019	0.131	-3.190	2.116	0.132	FALSE
Ability to smell	Parkinson's Disease	rs778138	2	61687214	-0.009	0.001	1.10E-14	-0.014	0.022	0.518	1.631	2.515	0.517	FALSE
Ability to smell	Parkinson's Disease	rs4538234	2	77703859	0.008	0.001	4.63E-11	0.013	0.029	0.645	1.756	3.804	0.644	FALSE
Ability to smell	Parkinson's Disease	rs60796561	2	86820350	0.010	0.002	2.13E-10	-0.016	0.028	0.573	-1.671	2.965	0.573	FALSE
Ability to smell	Parkinson's Disease	rs6715454	2	100480951	0.008	0.001	1.27E-12	-0.042	0.028	0.126	-5.392	3.518	0.125	FALSE
Ability to smell	Parkinson's Disease	rs1921622	2	102966067	0.006	0.001	3.00E-09	0.031	0.019	0.106	4.816	2.985	0.107	FALSE
Ability to smell	Parkinson's Disease	rs2198234	2	104159202	-0.012	0.001	5.73E-30	0.024	0.023	0.288	-1.917	1.804	0.288	FALSE
Ability to smell	Parkinson's Disease	rs59259939	2	115992239	-0.011	0.001	9.22E-24	0.001	0.021	0.950	-0.118	1.862	0.949	FALSE
Ability to smell	Parkinson's Disease	rs893434	2	127894757	0.008	0.001	5.03E-08	0.010	0.025	0.701	1.231	3.227	0.703	FALSE
Ability to smell	Parkinson's Disease	rs1712957	2	144505198	0.009	0.001	8.19E-15	0.031	0.028	0.272	3.618	3.291	0.272	FALSE
Ability to smell	Parkinson's Disease	rs9679427	2	148543968	-0.007	0.001	1.51E-09	0.056	0.022	0.010	-7.819	3.018	0.010	FALSE
Ability to smell	Parkinson's Disease	rs4577242	2	161221393	-0.006	0.001	1.45E-07	0.063	0.020	0.002	-10.832	3.415	0.002	FALSE
Ability to smell	Parkinson's Disease	rs10169534	2	162755829	-0.019	0.002	1.99E-15	-0.143	0.067	0.033	7.497	3.526	0.033	FALSE
Ability to smell	Parkinson's Disease	rs4273233	2	165117177	0.009	0.001	1.22E-11	-0.006	0.025	0.812	-0.679	2.839	0.811	FALSE
Ability to smell	Parkinson's Disease	rs13389333	2	176442641	0.006	0.001	4.67E-08	-0.035	0.025	0.156	-5.510	3.877	0.155	FALSE
Ability to smell	Parkinson's Disease	rs62180106	2	199629554	-0.007	0.001	1.06E-09	-0.028	0.020	0.159	4.008	2.847	0.159	FALSE
Ability to smell	Parkinson's Disease	rs717915	2	200714766	0.006	0.001	3.61E-08	0.076	0.022	0.001	12.132	3.550	0.001	FALSE
Ability to smell	Parkinson's Disease	rs2287431	2	211320288	-0.009	0.001	9.06E-14	0.021	0.030	0.495	-2.274	3.328	0.495	FALSE
Ability to smell	Parkinson's Disease	rs13030947	2	215051849	-0.006	0.001	1.14E-08	0.010	0.028	0.715	-1.593	4.353	0.714	FALSE
Ability to smell	Parkinson's Disease	rs6704768	2	233592501	-0.015	0.001	2.43E-42	-0.025	0.022	0.256	1.662	1.463	0.256	FALSE
Ability to smell	Parkinson's Disease	rs10865701	3	10431205	0.007	0.001	1.98E-10	-0.026	0.028	0.351	-3.562	3.825	0.352	FALSE

Ability to smell	Parkinson's Disease	rs9861687	3	20655103	0.008	0.001	1.18E-09	-0.025	0.031	0.414	-3.328	4.088	0.416	FALSE
Ability to smell	Parkinson's Disease	rs9847958	3	23361728	0.010	0.001	4.30E-18	0.077	0.029	0.007	7.739	2.890	0.007	FALSE
Ability to smell	Parkinson's Disease	rs1303630	3	25545500	-0.006	0.001	8.28E-08	0.011	0.027	0.681	-1.909	4.629	0.680	FALSE
Ability to smell	Parkinson's Disease	rs9870195	3	33923805	-0.009	0.001	1.16E-10	0.045	0.024	0.062	-5.286	2.830	0.062	FALSE
Ability to smell	Parkinson's Disease	rs6779394	3	49157771	0.009	0.001	5.37E-14	-0.037	0.020	0.062	-4.270	2.284	0.062	FALSE
Ability to smell	Parkinson's Disease	rs2710323	3	52815905	-0.009	0.001	1.35E-16	0.009	0.019	0.624	-1.017	2.078	0.625	FALSE
Ability to smell	Parkinson's Disease	rs11719771	3	65481987	-0.006	0.002	4.26E-05	0.062	0.038	0.100	-10.001	6.078	0.100	FALSE
Ability to smell	Parkinson's Disease	rs10511013	3	70853155	-0.007	0.001	4.65E-09	0.041	0.028	0.148	-6.241	4.319	0.149	FALSE
Ability to smell	Parkinson's Disease	rs9878602	3	71535338	0.007	0.001	3.58E-10	0.011	0.028	0.703	1.528	4.016	0.704	FALSE
Ability to smell	Parkinson's Disease	rs73138782	3	81976235	-0.021	0.003	5.76E-11	0.102	0.086	0.236	-4.795	4.038	0.235	FALSE
Ability to smell	Parkinson's Disease	rs12493563	3	85682087	-0.006	0.001	2.58E-07	0.009	0.020	0.653	-1.501	3.355	0.655	FALSE
Ability to smell	Parkinson's Disease	rs9881883	3	93804326	-0.015	0.003	4.36E-08	-0.034	0.069	0.620	2.370	4.781	0.620	FALSE
Ability to smell	Parkinson's Disease	rs13060816	3	107280486	0.007	0.001	6.26E-11	0.008	0.027	0.756	1.174	3.788	0.757	FALSE
Ability to smell	Parkinson's Disease	rs7611619	3	115100760	0.018	0.001	7.57E-56	-0.011	0.029	0.699	-0.621	1.606	0.699	FALSE
Ability to smell	Parkinson's Disease	rs35764849	3	115716166	0.012	0.001	2.62E-25	0.018	0.028	0.523	1.532	2.404	0.524	FALSE
Ability to smell	Parkinson's Disease	rs6440003	3	141094209	-0.007	0.001	2.18E-09	-0.003	0.019	0.862	0.499	2.886	0.863	FALSE
Ability to smell	Parkinson's Disease	rs73004622	3	147094819	0.012	0.002	2.19E-08	-0.061	0.054	0.261	-5.216	4.635	0.260	FALSE
Ability to smell	Parkinson's Disease	rs17283010	3	151097391	0.012	0.001	2.06E-24	-0.066	0.020	0.001	-5.740	1.691	0.001	FALSE
Ability to smell	Parkinson's Disease	rs4254636	3	178535820	-0.007	0.001	4.81E-08	-0.037	0.035	0.293	5.337	5.075	0.293	FALSE
Ability to smell	Parkinson's Disease	rs11545169	3	184020542	0.011	0.002	1.49E-13	0.043	0.026	0.095	3.882	2.325	0.095	FALSE
Ability to smell	Parkinson's Disease	rs338218	3	197027242	-0.009	0.001	4.52E-15	0.077	0.029	0.007	-8.645	3.228	0.007	FALSE
Ability to smell	Parkinson's Disease	rs1203850	4	2510481	-0.007	0.001	2.80E-11	-0.004	0.024	0.865	0.534	3.150	0.865	FALSE
Ability to smell	Parkinson's Disease	rs76522376	4	7291339	0.008	0.001	8.71E-11	0.037	0.031	0.228	4.740	3.929	0.228	FALSE
Ability to smell	Parkinson's Disease	rs12501390	4	10729363	0.007	0.001	7.82E-08	-0.029	0.023	0.206	-4.302	3.412	0.207	FALSE
Ability to smell	Parkinson's Disease	rs34811474	4	25408838	-0.010	0.001	2.55E-13	0.053	0.024	0.026	-5.075	2.275	0.026	FALSE
Ability to smell	Parkinson's Disease	rs11736548	4	28731737	-0.007	0.001	2.69E-08	-0.007	0.026	0.785	1.015	3.710	0.785	FALSE
Ability to smell	Parkinson's Disease	rs279826	4	46334209	-0.008	0.001	7.24E-13	0.035	0.028	0.206	-4.448	3.523	0.207	FALSE

Ability to smell	Parkinson's Disease	rs9992872	4	48728499	-0.008	0.001	1.35E-09	-0.013	0.029	0.647	1.593	3.466	0.646	FALSE
Ability to smell	Parkinson's Disease	rs10866117	4	62242432	0.006	0.001	1.29E-07	-0.016	0.029	0.579	-2.552	4.600	0.579	FALSE
Ability to smell	Parkinson's Disease	rs2614893	4	66895551	-0.006	0.001	1.18E-08	0.015	0.028	0.575	-2.459	4.391	0.575	FALSE
Ability to smell	Parkinson's Disease	rs58768853	4	67487670	-0.014	0.002	2.23E-17	-0.043	0.042	0.305	3.083	3.004	0.305	FALSE
Ability to smell	Parkinson's Disease	rs7688383	4	70481705	0.008	0.001	1.20E-11	-0.021	0.022	0.336	-2.725	2.830	0.335	FALSE
Ability to smell	Parkinson's Disease	rs6829629	4	80222954	0.006	0.001	9.68E-09	0.043	0.021	0.039	6.829	3.303	0.039	FALSE
Ability to smell	Parkinson's Disease	rs3756059	4	90757272	0.007	0.001	7.82E-11	-0.047	0.019	0.014	-6.536	2.651	0.014	FALSE
Ability to smell	Parkinson's Disease	rs10516927	4	94539295	-0.013	0.002	4.97E-14	-0.018	0.041	0.657	1.462	3.292	0.657	FALSE
Ability to smell	Parkinson's Disease	rs10017565	4	103861684	0.007	0.001	1.70E-10	-0.030	0.022	0.171	-4.264	3.105	0.170	FALSE
Ability to smell	Parkinson's Disease	rs2510652	4	105662306	0.007	0.001	8.03E-10	-0.022	0.029	0.457	-3.052	4.116	0.458	FALSE
Ability to smell	Parkinson's Disease	rs2647258	4	106248230	0.006	0.001	8.97E-07	0.013	0.020	0.530	2.297	3.672	0.532	FALSE
Ability to smell	Parkinson's Disease	rs62317264	4	130261585	-0.008	0.001	1.71E-14	-0.003	0.027	0.921	0.320	3.248	0.922	FALSE
Ability to smell	Parkinson's Disease	rs35400008	4	130548122	0.006	0.001	1.17E-07	0.038	0.030	0.210	5.901	4.705	0.210	FALSE
Ability to smell	Parkinson's Disease	rs5343	4	148464992	0.008	0.001	1.93E-11	0.012	0.029	0.672	1.586	3.753	0.673	FALSE
Ability to smell	Parkinson's Disease	rs62346281	4	171078722	-0.008	0.001	2.23E-14	0.033	0.021	0.108	-3.987	2.478	0.108	FALSE
Ability to smell	Parkinson's Disease	rs144183806	4	190535042	-0.032	0.005	3.30E-10	0.108	0.138	0.434	-3.376	4.317	0.434	FALSE
Ability to smell	Parkinson's Disease	rs4702351	5	6432839	0.007	0.001	5.63E-09	-0.059	0.029	0.040	-8.742	4.268	0.041	FALSE
Ability to smell	Parkinson's Disease	rs9885441	5	27045018	0.007	0.001	5.70E-11	0.008	0.020	0.675	1.171	2.787	0.674	FALSE
Ability to smell	Parkinson's Disease	rs11737962	5	64953081	-0.007	0.001	5.10E-09	0.030	0.027	0.273	-4.105	3.736	0.272	FALSE
Ability to smell	Parkinson's Disease	rs10041403	5	67805272	0.011	0.001	2.15E-13	-0.009	0.038	0.818	-0.808	3.510	0.818	FALSE
Ability to smell	Parkinson's Disease	rs7707104	5	89627079	-0.007	0.001	4.53E-10	0.046	0.021	0.029	-6.671	3.055	0.029	FALSE
Ability to smell	Parkinson's Disease	rs17669337	5	92187932	0.009	0.001	1.13E-15	0.020	0.028	0.482	2.202	3.135	0.482	FALSE
Ability to smell	Parkinson's Disease	rs13166639	5	92636386	-0.007	0.001	4.13E-09	0.024	0.030	0.432	-3.241	4.137	0.433	FALSE
Ability to smell	Parkinson's Disease	rs17314727	5	93228046	-0.016	0.001	8.90E-34	-0.040	0.033	0.233	2.492	2.090	0.233	FALSE
Ability to smell	Parkinson's Disease	rs2545751	5	112204207	-0.006	0.001	4.63E-05	-0.028	0.026	0.281	4.998	4.636	0.281	FALSE
Ability to smell	Parkinson's Disease	rs1425548	5	113961122	0.010	0.001	1.18E-16	-0.055	0.029	0.059	-5.605	2.971	0.059	FALSE
Ability to smell	Parkinson's Disease	rs2301579	5	131553340	0.010	0.001	6.44E-20	0.004	0.020	0.847	0.387	1.982	0.845	FALSE

Ability to smell	Parkinson's Disease	rs10065792	5	133038091	-0.006	0.001	3.83E-07	-0.039	0.026	0.139	6.112	4.132	0.139	FALSE
Ability to smell	Parkinson's Disease	rs11743259	5	137636706	-0.012	0.001	1.38E-18	0.032	0.025	0.193	-2.579	1.983	0.193	FALSE
Ability to smell	Parkinson's Disease	rs1623177	5	139460236	0.008	0.001	2.35E-12	0.018	0.030	0.544	2.213	3.643	0.544	FALSE
Ability to smell	Parkinson's Disease	rs6873273	5	143973969	0.008	0.001	2.81E-11	-0.008	0.032	0.797	-0.963	3.770	0.798	FALSE
Ability to smell	Parkinson's Disease	rs2962853	5	164300717	-0.005	0.001	6.66E-06	-0.001	0.029	0.986	0.094	5.525	0.986	FALSE
Ability to smell	Parkinson's Disease	rs61094404	5	164698686	-0.012	0.001	1.74E-26	-0.009	0.027	7.48E-01	0.747	2.327	7.48E-01	
Ability to smell	Parkinson's Disease	rs2617956	5	167416331	0.010	0.001	2.02E-16	0.049	0.031	0.115	4.883	3.102	0.115	FALSE
Ability to smell	Parkinson's Disease	rs36092039	5	177036256	-0.008	0.001	1.23E-12	0.003	0.023	0.903	-0.340	2.769	0.902	FALSE
Ability to smell	Parkinson's Disease	rs7728341	5	179524425	0.006	0.001	2.03E-08	0.006	0.027	0.821	1.007	4.452	0.821	FALSE
Ability to smell	Parkinson's Disease	rs28371453	6	16846022	-0.017	0.003	2.13E-08	-0.143	0.077	0.062	8.512	4.569	0.062	FALSE
Ability to smell	Parkinson's Disease	rs12665528	6	16936032	-0.007	0.001	1.08E-08	0.038	0.031	0.226	-5.227	4.315	0.226	FALSE
Ability to smell	Parkinson's Disease	rs9380526	6	35658327	-0.007	0.001	6.08E-09	-0.033	0.024	0.168	4.870	3.541	0.169	FALSE
Ability to smell	Parkinson's Disease	rs10498754	6	43156160	-0.016	0.002	5.62E-14	0.007	0.042	0.862	-0.458	2.635	0.862	FALSE
Ability to smell	Parkinson's Disease	rs148736595	6	43601515	0.021	0.004	1.65E-06	0.076	0.086	0.376	3.676	4.149	0.376	FALSE
Ability to smell	Parkinson's Disease	rs17767336	6	90800438	0.008	0.001	4.83E-08	0.041	0.037	0.270	5.100	4.634	0.271	FALSE
Ability to smell	Parkinson's Disease	rs41430945	6	94555154	-0.005	0.001	1.18E-06	0.002	0.021	0.939	-0.300	3.951	0.940	FALSE
Ability to smell	Parkinson's Disease	rs1933720	6	98297832	-0.012	0.001	7.86E-27	0.029	0.029	0.316	-2.351	2.343	0.316	FALSE
Ability to smell	Parkinson's Disease	rs454568	6	105442637	0.007	0.001	2.66E-11	0.008	0.021	0.697	1.090	2.794	0.696	FALSE
Ability to smell	Parkinson's Disease	rs4128270	6	115910044	0.007	0.001	9.80E-10	-0.008	0.021	0.703	-1.184	3.093	0.702	FALSE
Ability to smell	Parkinson's Disease	rs9401850	6	126151000	-0.004	0.001	4.39E-04	0.027	0.023	0.235	-6.814	5.746	0.236	FALSE
Ability to smell	Parkinson's Disease	rs13197257	6	128333682	-0.012	0.001	5.01E-21	-0.008	0.032	0.793	0.714	2.717	0.793	FALSE
Ability to smell	Parkinson's Disease	rs13214277	6	146136125	-0.007	0.001	7.64E-11	-0.006	0.019	0.758	0.802	2.614	0.759	FALSE
Ability to smell	Parkinson's Disease	rs9456784	6	162934970	0.007	0.001	2.92E-09	-0.042	0.029	0.141	-6.354	4.312	0.141	FALSE
Ability to smell	Parkinson's Disease	rs7383874	7	1714844	-0.010	0.001	9.47E-17	-0.014	0.030	0.639	1.408	3.005	0.639	FALSE
Ability to smell	Parkinson's Disease	rs6973573	7	5279131	0.004	0.001	5.03E-05	-0.027	0.031	0.389	-5.936	6.899	0.390	FALSE
Ability to smell	Parkinson's Disease	rs6971441	7	14801502	0.010	0.002	1.21E-09	-0.006	0.030	0.833	-0.628	2.956	0.832	FALSE
Ability to smell	Parkinson's Disease	rs858279	7	23251005	0.006	0.001	7.83E-09	-0.086	0.020	0.000	-13.329	3.072	0.000	FALSE

Ability to smell	Parkinson's Disease	rs10228058	7	33047249	0.007	0.001	2.24E-10	-0.056	0.028	0.042	-7.997	3.927	0.042	FALSE
Ability to smell	Parkinson's Disease	rs6943073	7	49860604	0.007	0.001	7.97E-09	-0.034	0.026	0.192	-4.738	3.644	0.193	FALSE
Ability to smell	Parkinson's Disease	rs58148428	7	52870435	0.008	0.001	2.64E-08	-0.033	0.035	0.339	-4.198	4.400	0.340	FALSE
Ability to smell	Parkinson's Disease	rs2968532	7	71787692	0.012	0.001	1.47E-26	-0.019	0.022	0.379	-1.637	1.857	0.378	FALSE
Ability to smell	Parkinson's Disease	rs13226650	7	73017005	-0.008	0.001	2.20E-09	-0.028	0.026	0.274	3.334	3.058	0.276	FALSE
Ability to smell	Parkinson's Disease	rs4529405	7	84405422	-0.007	0.001	2.47E-09	-0.006	0.022	0.773	0.966	3.343	0.773	FALSE
Ability to smell	Parkinson's Disease	rs858502	7	99843353	-0.007	0.001	1.71E-09	-0.011	0.020	0.587	1.647	3.037	0.588	FALSE
Ability to smell	Parkinson's Disease	rs71582886	7	103390132	0.020	0.003	1.55E-09	0.039	0.065	0.552	1.942	3.269	0.552	FALSE
Ability to smell	Parkinson's Disease	rs35307180	7	108288453	-0.006	0.001	1.53E-07	-0.001	0.027	0.984	0.102	4.627	0.982	FALSE
Ability to smell	Parkinson's Disease	rs13246732	7	113775765	-0.008	0.001	1.46E-12	-0.003	0.031	0.929	0.320	3.614	0.929	FALSE
Ability to smell	Parkinson's Disease	rs7349958	7	114251286	0.014	0.002	6.67E-16	-0.023	0.044	0.606	-1.661	3.212	0.605	FALSE
Ability to smell	Parkinson's Disease	rs13239186	7	117510621	-0.012	0.001	1.26E-22	-0.051	0.029	0.078	4.378	2.476	0.077	FALSE
Ability to smell	Parkinson's Disease	rs3808150	7	126386068	-0.005	0.001	9.30E-06	0.010	0.021	0.625	-2.068	4.217	0.624	FALSE
Ability to smell	Parkinson's Disease	rs12154492	7	127584722	-0.010	0.001	1.47E-17	0.007	0.021	0.755	-0.640	2.045	0.754	FALSE
Ability to smell	Parkinson's Disease	rs6956399	7	133305729	0.010	0.001	3.11E-12	-0.005	0.033	0.889	-0.493	3.507	0.888	FALSE
Ability to smell	Parkinson's Disease	rs59768128	8	675703	-0.005	0.001	3.95E-05	-0.018	0.032	0.575	3.802	6.769	0.574	FALSE
Ability to smell	Parkinson's Disease	rs13269741	8	10159818	0.010	0.001	6.81E-17	0.034	0.029	0.232	3.285	2.749	0.232	FALSE
Ability to smell	Parkinson's Disease	rs7460852	8	10295112	-0.008	0.002	4.50E-08	-0.032	0.031	0.306	3.852	3.767	0.307	FALSE
Ability to smell	Parkinson's Disease	rs12544554	8	23105268	0.007	0.001	1.63E-09	-0.010	0.023	0.654	-1.498	3.353	0.655	FALSE
Ability to smell	Parkinson's Disease	rs62502022	8	25315837	-0.021	0.003	1.98E-16	-0.004	0.070	0.950	0.213	3.405	0.950	FALSE
Ability to smell	Parkinson's Disease	rs7016874	8	30915627	-0.006	0.001	3.07E-08	-0.044	0.022	0.042	6.969	3.422	0.042	FALSE
Ability to smell	Parkinson's Disease	rs4262318	8	52678558	0.008	0.001	5.97E-11	-0.034	0.030	0.249	-4.310	3.743	0.250	FALSE
Ability to smell	Parkinson's Disease	rs1217353	8	64596597	-0.016	0.002	1.91E-10	0.023	0.063	0.712	-1.484	4.026	0.712	FALSE
Ability to smell	Parkinson's Disease	rs2612607	8	65340163	0.010	0.001	3.72E-14	0.027	0.039	0.501	2.538	3.774	0.501	FALSE
Ability to smell	Parkinson's Disease	rs6992179	8	75142288	-0.010	0.001	1.14E-14	-0.009	0.032	0.773	0.940	3.275	0.774	FALSE
Ability to smell	Parkinson's Disease	rs7002652	8	93282549	0.006	0.001	4.68E-09	-0.062	0.028	0.023	-9.657	4.270	0.024	FALSE
Ability to smell	Parkinson's Disease	rs6998822	8	94533739	-0.009	0.002	5.10E-09	-0.031	0.037	0.402	3.426	4.087	0.402	FALSE

Ability to smell	Parkinson's Disease	rs4242551	8	114171732	0.006	0.001	9.26E-09	0.030	0.022	0.170	4.729	3.443	0.170	FALSE
Ability to smell	Parkinson's Disease	rs68018407	8	117446155	-0.012	0.002	1.26E-09	-0.017	0.047	0.719	1.444	4.008	0.719	FALSE
Ability to smell	Parkinson's Disease	rs4565426	8	130639222	0.008	0.001	6.79E-11	-0.026	0.025	0.302	-3.284	3.183	0.302	FALSE
Ability to smell	Parkinson's Disease	rs1158570	8	131331073	0.008	0.001	3.29E-14	-0.053	0.028	0.056	-6.384	3.342	0.056	FALSE
Ability to smell	Parkinson's Disease	rs2467997	8	133859871	0.008	0.001	7.96E-11	0.003	0.025	0.918	0.320	3.048	0.917	FALSE
Ability to smell	Parkinson's Disease	rs11787216	8	142615222	0.008	0.001	3.07E-13	-0.003	0.029	0.911	-0.379	3.435	0.912	FALSE
Ability to smell	Parkinson's Disease	rs4072917	8	143300279	-0.007	0.001	1.44E-11	0.006	0.022	0.792	-0.780	2.972	0.793	FALSE
Ability to smell	Parkinson's Disease	rs558706	9	3967879	-0.008	0.001	2.09E-10	-0.030	0.031	0.335	3.723	3.860	0.335	FALSE
Ability to smell	Parkinson's Disease	rs4742127	9	5860957	-0.008	0.001	2.74E-10	-0.060	0.031	0.051	7.691	3.936	0.051	FALSE
Ability to smell	Parkinson's Disease	rs992969	9	6209697	-0.010	0.001	4.11E-14	0.019	0.022	0.379	-2.040	2.323	0.380	FALSE
Ability to smell	Parkinson's Disease	rs1494343	9	14735694	-0.006	0.001	3.71E-07	-0.015	0.021	0.484	2.390	3.417	0.484	FALSE
Ability to smell	Parkinson's Disease	rs4567133	9	22606560	0.009	0.001	9.08E-11	0.030	0.036	0.398	3.275	3.876	0.398	FALSE
Ability to smell	Parkinson's Disease	rs7468681	9	23347917	0.007	0.001	4.79E-09	0.045	0.030	0.134	6.372	4.253	0.134	FALSE
Ability to smell	Parkinson's Disease	rs7047853	9	80115740	-0.006	0.001	2.20E-08	0.036	0.028	0.191	-5.889	4.502	0.191	FALSE
Ability to smell	Parkinson's Disease	rs3087979	9	111781779	-0.007	0.001	8.19E-09	-0.049	0.022	0.026	6.998	3.136	0.026	FALSE
Ability to smell	Parkinson's Disease	rs7868227	9	124738167	-0.006	0.001	4.50E-09	0.016	0.027	0.570	-2.411	4.234	0.569	FALSE
Ability to smell	Parkinson's Disease	rs700061	9	125824478	0.011	0.002	5.59E-11	-0.022	0.031	0.467	-2.099	2.889	0.468	FALSE
Ability to smell	Parkinson's Disease	rs7039136	9	129255630	-0.008	0.001	3.71E-08	0.024	0.035	0.494	-3.095	4.520	0.493	FALSE
Ability to smell	Parkinson's Disease	rs78706681	10	596939	-0.016	0.003	1.04E-09	-0.050	0.067	0.451	3.240	4.294	0.451	FALSE
Ability to smell	Parkinson's Disease	rs7907344	10	10958521	0.007	0.001	1.00E-10	-0.025	0.023	0.292	-3.444	3.261	0.291	FALSE
Ability to smell	Parkinson's Disease	rs687094	10	28972803	-0.008	0.002	3.54E-08	-0.073	0.039	0.061	8.768	4.679	0.061	FALSE
Ability to smell	Parkinson's Disease	rs2246585	10	53763998	-0.008	0.001	2.75E-10	0.024	0.031	0.441	-3.031	3.936	0.441	FALSE
Ability to smell	Parkinson's Disease	rs3099370	10	61696597	-0.008	0.001	7.12E-13	0.043	0.028	0.125	-5.315	3.464	0.125	FALSE
Ability to smell	Parkinson's Disease	rs10994386	10	62260544	0.009	0.002	2.03E-07	0.038	0.045	0.404	4.354	5.215	0.404	FALSE
Ability to smell	Parkinson's Disease	rs2593163	10	73585526	0.010	0.002	8.62E-09	0.014	0.033	0.673	1.341	3.170	0.672	FALSE
Ability to smell	Parkinson's Disease	rs11816241	10	94643656	-0.009	0.001	1.31E-13	0.016	0.023	0.494	-1.718	2.517	0.495	FALSE
Ability to smell	Parkinson's Disease	rs2305191	10	103560333	0.009	0.001	7.88E-16	-0.048	0.022	0.026	-5.339	2.399	0.026	FALSE

Ability to smell	Parkinson's Disease	rs2130519	10	106566082	0.009	0.002	2.32E-08	-0.010	0.041	0.804	-1.116	4.485	0.804	FALSE
Ability to smell	Parkinson's Disease	rs2946993	10	126728403	0.006	0.001	4.24E-08	-0.013	0.019	0.509	-2.111	3.208	0.511	FALSE
Ability to smell	Parkinson's Disease	rs17153017	10	127155393	0.009	0.001	4.48E-10	0.036	0.034	0.291	4.166	3.946	0.291	FALSE
Ability to smell	Parkinson's Disease	rs7093416	10	135499157	-0.006	0.001	6.56E-06	0.027	0.039	0.495	-4.160	6.099	0.495	FALSE
Ability to smell	Parkinson's Disease	rs1466234	11	8398847	0.007	0.001	1.21E-08	0.005	0.021	0.817	0.747	3.233	0.817	FALSE
Ability to smell	Parkinson's Disease	rs1544861	11	10679441	0.007	0.001	8.46E-09	-0.023	0.020	0.253	-3.507	3.071	0.253	FALSE
Ability to smell	Parkinson's Disease	rs12808129	11	13246222	0.009	0.001	1.43E-15	-0.002	0.027	0.943	-0.216	3.065	0.944	FALSE
Ability to smell	Parkinson's Disease	rs11606078	11	30541533	-0.010	0.001	2.62E-14	0.022	0.026	0.403	-2.202	2.631	0.402	FALSE
Ability to smell	Parkinson's Disease	rs2071164	11	31833542	0.013	0.002	1.11E-17	-0.033	0.039	0.400	-2.476	2.944	0.400	FALSE
Ability to smell	Parkinson's Disease	rs12290967	11	41261033	0.005	0.001	2.86E-06	0.016	0.021	0.447	3.135	4.108	0.445	FALSE
Ability to smell	Parkinson's Disease	rs2047812	11	48162042	0.010	0.001	7.39E-12	-0.003	0.024	0.894	-0.333	2.527	0.895	FALSE
Ability to smell	Parkinson's Disease	rs118093058	11	62413673	-0.010	0.002	5.19E-10	0.051	0.032	0.112	-5.097	3.214	0.113	FALSE
Ability to smell	Parkinson's Disease	rs7119426	11	66649612	-0.007	0.001	2.79E-09	-0.010	0.021	0.646	1.317	2.867	0.646	FALSE
Ability to smell	Parkinson's Disease	rs2374577	11	84643427	0.008	0.001	3.50E-13	-0.025	0.027	0.340	-3.134	3.295	0.342	FALSE
Ability to smell	Parkinson's Disease	rs7938634	11	85845473	-0.004	0.001	1.49E-03	0.051	0.020	0.011	-14.249	5.577	0.011	FALSE
Ability to smell	Parkinson's Disease	rs2155077	11	90389335	0.007	0.001	1.23E-08	0.002	0.029	0.937	0.338	4.326	0.938	FALSE
Ability to smell	Parkinson's Disease	rs2170719	11	104012767	-0.007	0.001	3.51E-09	-0.007	0.028	0.801	1.067	4.237	0.801	FALSE
Ability to smell	Parkinson's Disease	rs2187388	11	111387116	-0.005	0.001	2.15E-05	0.006	0.029	0.840	-1.175	5.873	0.841	FALSE
Ability to smell	Parkinson's Disease	rs2105619	11	111825253	0.008	0.001	5.33E-13	-0.010	0.020	0.598	-1.264	2.395	0.598	FALSE
Ability to smell	Parkinson's Disease	rs3802851	11	112912550	0.008	0.001	9.39E-10	-0.013	0.033	0.687	-1.761	4.362	0.686	FALSE
Ability to smell	Parkinson's Disease	rs56094217	11	115173755	0.009	0.002	2.53E-08	0.009	0.039	0.824	0.997	4.493	0.824	FALSE
Ability to smell	Parkinson's Disease	rs1065	11	132273416	0.007	0.001	4.93E-08	-0.029	0.022	0.191	-4.382	3.355	0.192	FALSE
Ability to smell	Parkinson's Disease	rs4388955	12	926702	0.009	0.001	1.54E-12	-0.019	0.025	0.434	-2.171	2.770	0.433	FALSE
Ability to smell	Parkinson's Disease	rs1379511	12	13581604	0.005	0.001	1.80E-05	0.036	0.028	0.199	7.532	5.853	0.198	FALSE
Ability to smell	Parkinson's Disease	rs10840683	12	17413963	0.007	0.001	1.72E-11	0.035	0.028	0.201	4.726	3.692	0.201	FALSE
Ability to smell	Parkinson's Disease	rs11049380	12	28313837	-0.011	0.001	2.26E-19	0.005	0.027	0.854	-0.436	2.369	0.854	FALSE
Ability to smell	Parkinson's Disease	rs7137139	12	39364013	0.007	0.001	2.50E-11	-0.008	0.021	0.718	-1.028	2.826	0.716	FALSE

Ability to smell	Parkinson's Disease	rs1366024	12	46313638	-0.007	0.001	2.16E-09	-0.054	0.019	0.005	8.229	2.931	0.005	FALSE
Ability to smell	Parkinson's Disease	rs773125	12	56394954	-0.007	0.001	9.70E-11	0.005	0.020	0.794	-0.726	2.780	0.794	FALSE
Ability to smell	Parkinson's Disease	rs75231257	12	58094274	-0.016	0.003	6.33E-09	-0.015	0.063	0.818	0.909	3.942	0.818	FALSE
Ability to smell	Parkinson's Disease	rs249623	12	98192058	0.007	0.001	4.31E-09	-0.022	0.031	0.476	-3.039	4.266	0.476	FALSE
Ability to smell	Parkinson's Disease	rs11112945	12	106739049	0.008	0.001	1.56E-09	0.018	0.033	0.586	2.259	4.132	0.584	FALSE
Ability to smell	Parkinson's Disease	rs3764002	12	108618630	-0.013	0.001	1.46E-24	0.053	0.022	0.015	-4.053	1.669	0.015	FALSE
Ability to smell	Parkinson's Disease	rs12823540	12	110308549	0.007	0.001	4.28E-09	0.019	0.028	0.488	2.890	4.176	0.489	FALSE
Ability to smell	Parkinson's Disease	rs1265565	12	111715197	-0.009	0.001	4.36E-14	0.006	0.024	0.821	-0.614	2.704	0.820	FALSE
Ability to smell	Parkinson's Disease	rs7133013	12	121368468	0.011	0.002	3.26E-11	0.017	0.031	0.575	1.595	2.842	0.575	FALSE
Ability to smell	Parkinson's Disease	rs11246795	12	132232079	-0.025	0.004	2.21E-08	0.002	0.076	0.985	-0.060	3.056	0.984	FALSE
Ability to smell	Parkinson's Disease	rs7137653	12	133401094	-0.008	0.001	3.49E-10	-0.001	0.022	0.968	0.111	2.702	0.967	TRUE
Ability to smell	Parkinson's Disease	rs1606182	13	31763090	-0.009	0.001	3.22E-12	0.014	0.027	0.604	-1.532	2.964	0.605	FALSE
Ability to smell	Parkinson's Disease	rs7996330	13	53893310	0.010	0.001	5.59E-18	0.023	0.023	0.310	2.385	2.343	0.309	FALSE
Ability to smell	Parkinson's Disease	rs9316841	13	56775768	-0.008	0.001	9.39E-10	0.013	0.030	0.668	-1.515	3.540	0.669	FALSE
Ability to smell	Parkinson's Disease	rs7986422	13	66416009	-0.008	0.001	1.61E-12	-0.008	0.023	0.732	1.032	2.993	0.730	FALSE
Ability to smell	Parkinson's Disease	rs9564497	13	69388108	0.008	0.001	3.87E-08	0.026	0.038	0.499	3.112	4.600	0.499	FALSE
Ability to smell	Parkinson's Disease	rs9517578	13	99712485	-0.008	0.001	8.85E-12	-0.054	0.024	0.027	6.640	3.005	0.027	FALSE
Ability to smell	Parkinson's Disease	rs7335993	13	100739135	0.007	0.001	4.88E-09	0.013	0.029	0.647	1.965	4.315	0.649	FALSE
Ability to smell	Parkinson's Disease	rs1283200	13	101322875	-0.008	0.001	2.13E-13	0.008	0.023	0.738	-0.919	2.745	0.738	FALSE
Ability to smell	Parkinson's Disease	rs111300896	14	22068322	-0.011	0.002	2.58E-09	0.003	0.049	0.958	-0.225	4.381	0.959	FALSE
Ability to smell	Parkinson's Disease	rs7144135	14	22138328	-0.028	0.001	3.42E-84	-0.018	0.024	0.457	0.652	0.877	0.457	TRUE
Ability to smell	Parkinson's Disease	rs1051695	14	33293122	-0.006	0.001	4.88E-09	0.006	0.019	0.756	-0.900	2.903	0.756	FALSE
Ability to smell	Parkinson's Disease	rs1018466	14	37123250	-0.006	0.001	9.16E-09	-0.024	0.027	0.3781	3.828	4.352	0.3781	FALSE
Ability to smell	Parkinson's Disease	rs6571932	14	39853750	-0.007	0.001	7.36E-11	-0.009	0.021	0.654	1.270	2.844	0.655	FALSE
Ability to smell	Parkinson's Disease	rs10143158	14	42428658	0.009	0.001	1.39E-09	-0.055	0.036	0.131	-6.422	4.250	0.131	FALSE
Ability to smell	Parkinson's Disease	rs4902026	14	61557433	0.008	0.001	4.39E-11	0.021	0.023	0.354	2.633	2.831	0.352	FALSE
Ability to smell	Parkinson's Disease	rs10145001	14	64948940	-0.007	0.001	1.48E-10	-0.004	0.021	0.840	0.566	2.777	0.838	FALSE

Ability to smell	Parkinson's Disease	rs41228	14	71824530	0.007	0.001	2.28E-09	0.025	0.026	0.331	3.412	3.508	0.331	FALSE
Ability to smell	Parkinson's Disease	rs1532729	14	79495343	0.007	0.001	1.86E-10	-0.032	0.029	0.263	-4.456	3.974	0.262	FALSE
Ability to smell	Parkinson's Disease	rs11160618	14	101512972	0.007	0.001	2.76E-07	-0.004	0.028	0.879	-0.605	3.969	0.879	FALSE
Ability to smell	Parkinson's Disease	rs3803286	14	103246470	0.008	0.001	1.27E-12	-0.002	0.023	0.917	-0.291	2.842	0.918	FALSE
Ability to smell	Parkinson's Disease	rs6606909	15	27241301	-0.006	0.001	1.70E-08	0.022	0.026	0.412	-3.502	4.261	0.411	FALSE
Ability to smell	Parkinson's Disease	rs12917189	15	43023482	-0.008	0.001	2.12E-09	-0.051	0.023	0.024	6.370	2.826	0.024	FALSE
Ability to smell	Parkinson's Disease	rs1693493	15	52774924	-0.008	0.001	1.63E-10	0.066	0.032	0.039	-8.003	3.880	0.039	FALSE
Ability to smell	Parkinson's Disease	rs2243504	15	57597280	-0.009	0.001	9.36E-15	-0.005	0.021	0.802	0.598	2.402	0.804	FALSE
Ability to smell	Parkinson's Disease	rs4777183	15	70004775	0.006	0.001	6.27E-09	0.003	0.020	0.881	0.471	3.095	0.879	FALSE
Ability to smell	Parkinson's Disease	rs113931022	15	78901113	-0.008	0.001	5.25E-11	0.026	0.020	0.189	-3.414	2.593	0.188	FALSE
Ability to smell	Parkinson's Disease	rs2759310	15	81015065	-0.007	0.001	1.76E-09	0.065	0.029	0.025	-9.340	4.170	0.025	FALSE
Ability to smell	Parkinson's Disease	rs4778984	15	82508746	-0.005	0.001	1.19E-04	-0.042	0.024	0.075	8.696	4.886	0.075	FALSE
Ability to smell	Parkinson's Disease	rs12149498	16	620709	-0.010	0.001	3.22E-13	-0.020	0.024	0.392	2.064	2.411	0.392	FALSE
Ability to smell	Parkinson's Disease	rs17648524	16	7459683	0.006	0.001	1.24E-08	-0.034	0.029	0.240	-5.189	4.415	0.240	FALSE
Ability to smell	Parkinson's Disease	rs11647633	16	12670088	-0.009	0.002	3.93E-08	0.020	0.030	0.501	-2.332	3.475	0.502	FALSE
Ability to smell	Parkinson's Disease	rs11641598	16	15816881	-0.009	0.001	6.27E-09	0.043	0.038	0.251	-4.958	4.315	0.251	FALSE
Ability to smell	Parkinson's Disease	rs11646496	16	20200349	0.007	0.001	5.56E-07	0.133	0.037	0.000	18.602	5.179	0.000	FALSE
Ability to smell	Parkinson's Disease	rs4149391	16	28617971	0.009	0.001	2.51E-09	-0.034	0.026	0.195	-3.983	3.075	0.195	FALSE
Ability to smell	Parkinson's Disease	rs56391038	16	51611887	-0.012	0.001	2.05E-18	-0.014	0.035	0.697	1.096	2.817	0.697	FALSE
Ability to smell	Parkinson's Disease	rs12600322	16	52186525	0.011	0.002	1.20E-08	0.029	0.050	0.566	2.537	4.422	0.566	FALSE
Ability to smell	Parkinson's Disease	rs3784895	16	57941397	-0.008	0.001	1.39E-12	0.043	0.024	0.069	-5.238	2.876	0.069	FALSE
Ability to smell	Parkinson's Disease	rs7196853	16	67560613	0.013	0.002	2.45E-12	0.024	0.030	0.429	1.915	2.426	0.430	FALSE
Ability to smell	Parkinson's Disease	rs12924872	16	69552215	0.008	0.001	2.99E-14	0.023	0.020	0.243	2.726	2.332	0.242	FALSE
Ability to smell	Parkinson's Disease	rs9935325	16	71093625	-0.007	0.001	4.91E-10	-0.020	0.021	0.341	2.876	3.017	0.340	FALSE
Ability to smell	Parkinson's Disease	rs9923598	16	87462814	0.009	0.001	3.14E-09	0.012	0.025	0.626	1.428	2.937	0.627	FALSE
Ability to smell	Parkinson's Disease	rs71368508	17	4521473	0.025	0.004	7.64E-10	-1.49E-02	0.081	0.854	-0.594	3.227	0.854	FALSE
Ability to smell	Parkinson's Disease	rs72829457	17	7568327	-0.012	0.002	5.42E-11	0.033	0.033	0.317	-2.775	2.775	0.317	FALSE

Ability to smell	Parkinson's Disease	rs1638526	17	19848450	0.010	0.001	2.50E-14	0.007	0.023	0.756	0.746	2.396	0.755	FALSE
Ability to smell	Parkinson's Disease	rs876493	17	37824545	-0.008	0.001	1.23E-12	-0.021	0.020	0.305	2.644	2.567	0.303	FALSE
Ability to smell	Parkinson's Disease	rs7225002	17	44189067	0.018	0.001	2.94E-59	-0.081	0.023	4.91E-04	-4.481	1.284	4.84E-04	FALSE
Ability to smell	Parkinson's Disease	rs28412876	17	47454515	-0.009	0.001	7.31E-16	-0.007	0.020	0.738	0.734	2.190	0.738	FALSE
Ability to smell	Parkinson's Disease	rs2079270	17	48395982	0.006	0.001	8.91E-08	0.024	0.030	0.422	3.979	4.949	0.421	FALSE
Ability to smell	Parkinson's Disease	rs7405987	17	66021394	0.010	0.001	7.17E-14	-0.007	0.031	0.825	-0.690	3.117	0.825	FALSE
Ability to smell	Parkinson's Disease	rs9905914	17	77772068	-0.008	0.001	4.59E-13	-0.013	0.026	0.611	1.658	3.267	0.612	FALSE
Ability to smell	Parkinson's Disease	rs56278119	17	79045766	0.006	0.001	1.47E-06	-0.013	0.025	0.611	-2.105	4.127	0.610	FALSE
Ability to smell	Parkinson's Disease	rs8075338	17	79530332	-0.009	0.001	9.94E-15	0.010	0.020	0.601	-1.218	2.331	0.601	FALSE
Ability to smell	Parkinson's Disease	rs303757	18	21078716	0.011	0.001	1.29E-21	-0.008	0.020	0.704	-0.708	1.851	0.702	FALSE
Ability to smell	Parkinson's Disease	rs331992	18	24543117	0.008	0.001	3.85E-14	-0.035	0.028	0.209	-4.174	3.325	0.209	FALSE
Ability to smell	Parkinson's Disease	rs4799358	18	31586132	0.006	0.001	1.16E-08	-0.047	0.023	0.043	-7.488	3.704	0.043	FALSE
Ability to smell	Parkinson's Disease	rs2361050	18	42466387	0.008	0.001	1.53E-10	-0.058	0.023	0.012	-7.634	3.051	0.012	FALSE
Ability to smell	Parkinson's Disease	rs17766830	18	44040660	-0.007	0.001	8.78E-09	0.033	0.027	0.232	-4.362	3.651	0.232	FALSE
Ability to smell	Parkinson's Disease	rs1011339	18	52821063	0.006	0.001	1.52E-08	0.016	0.019	0.392	2.626	3.061	0.391	FALSE
Ability to smell	Parkinson's Disease	rs4144686	18	53251725	0.012	0.001	3.53E-15	-0.006	0.037	0.877	-0.485	3.160	0.878	FALSE
Ability to smell	Parkinson's Disease	rs73482000	18	72996833	-0.009	0.001	5.18E-14	0.001	0.020	0.968	-0.091	2.252	0.968	FALSE
Ability to smell	Parkinson's Disease	rs12457322	18	77583797	0.007	0.001	4.86E-09	-0.049	0.026	0.063	-6.908	3.718	0.063	FALSE
Ability to smell	Parkinson's Disease	rs62109933	19	13285508	-0.007	0.001	2.24E-08	0.038	0.037	0.306	-5.117	4.983	0.304	FALSE
Ability to smell	Parkinson's Disease	rs33431	19	30939989	0.006	0.001	1.29E-08	-0.001	0.021	0.952	-0.187	3.244	0.954	FALSE
Ability to smell	Parkinson's Disease	rs4805579	19	31052274	-0.007	0.001	1.75E-08	0.069	0.030	0.020	-10.384	4.457	0.020	FALSE
Ability to smell	Parkinson's Disease	rs358544	19	32993657	0.007	0.001	1.00E-05	-0.040	0.038	0.291	-6.174	5.851	0.291	FALSE
Ability to smell	Parkinson's Disease	rs113408217	19	36976870	-0.011	0.001	6.33E-18	0.014	0.027	0.612	-1.211	2.387	0.612	FALSE
Ability to smell	Parkinson's Disease	rs187361489	19	42833279	-0.027	0.006	5.57E-06	0.065	0.168	0.697	-2.379	6.103	0.697	FALSE
Ability to smell	Parkinson's Disease	rs10408179	19	46157004	0.007	0.001	4.32E-11	-0.052	0.022	0.018	-7.111	2.995	0.018	FALSE
Ability to smell	Parkinson's Disease	rs28590228	19	47581242	0.007	0.001	7.88E-09	0.003	0.020	0.893	0.399	3.001	0.894	FALSE
Ability to smell	Parkinson's Disease	rs432426	19	58780131	0.008	0.001	1.42E-13	-0.025	0.019	0.196	-3.004	2.323	0.196	FALSE

Ability to smell	Parkinson's Disease	rs17776374	20	17298146	0.007	0.001	3.01E-08	-0.012	0.033	0.713	-1.652	4.494	0.713	FALSE
Ability to smell	Parkinson's Disease	rs6058569	20	30817819	0.007	0.001	7.85E-11	-0.009	0.022	0.682	-1.254	3.065	0.682	FALSE
Ability to smell	Parkinson's Disease	rs1060402	20	49205095	-0.007	0.001	7.47E-10	-0.023	0.028	0.413	3.357	4.097	0.413	FALSE
Ability to smell	Parkinson's Disease	rs6518100	21	22717430	0.007	0.001	2.48E-10	-0.039	0.028	0.163	-5.547	3.976	0.163	FALSE
Ability to smell	Parkinson's Disease	rs9975562	21	40678968	0.009	0.001	5.47E-15	-0.008	0.021	0.714	-0.879	2.405	0.715	FALSE
Ability to smell	Parkinson's Disease	rs406388	22	18226997	0.008	0.001	7.12E-09	-0.013	0.037	0.736	-1.514	4.482	0.736	FALSE
Ability to smell	Parkinson's Disease	rs1138120	22	30229954	0.011	0.001	5.50E-23	-0.010	0.022	0.648	-0.875	1.919	0.649	FALSE
Ability to smell	Parkinson's Disease	rs133908	22	44522741	0.007	0.001	1.40E-08	0.027	0.029	0.351	4.183	4.474	0.350	FALSE
Ability to smell	Parkinson's Disease	rs12160976	22	46438246	-0.009	0.001	7.78E-14	0.019	0.022	0.388	-2.181	2.526	0.388	FALSE
Ability to smell	Parkinson's Disease	rs79966207	22	50722408	0.010	0.002	2.55E-10	-3.00E-04	0.026	0.992	-0.031	2.644	0.991	FALSE

Supplementary Table 6 – Cohort composition of the 23andMe reference panel. CHM syndip - Syndip benchmark dataset; HGDP - Human Genome Diversity Project; SGDP - Simons Genome Diversity Project.

**Cohort**	**Origin**	**Samples**	**Approximate ancestry**
AFAM	Internal	2,389	African American
AJ	Internal	553	Ashkenazi Jewish
Parkinson's cohort	Internal	2,721	European
Latino	Internal	1,961	Latino
LRRK2 carriers	Internal	303	European
1000 Genomes	External	2,493	Diverse
CHM syndip	External	1	European
GTEx v8	External	838	Diverse (USA)
HGDP	External	770	Diverse
SGDP	External	188	Diverse
**Total**		**12,217**	
